# Long‐Term Maintenance of Complex Chromosomal Inversion Polymorphism in *Drosophila mediopunctata*

**DOI:** 10.1002/ece3.70443

**Published:** 2024-10-27

**Authors:** Fabiana Uno, Felipe Bastos Rocha, Louis Bernard Klaczko

**Affiliations:** ^1^ Departamento de Genética Universidade Federal do Rio de Janeiro (UFRJ) Rio de Janeiro RJ Brazil; ^2^ Departamento de Genética ICBS, Universidade Federal Rural Do Rio de Janeiro (UFRRJ) Seropedica Brazil; ^3^ Departamento de Genética, Evolução, Microbiologia e Imunologia Inst. de Biologia, Universidade Estadual de Campinas (UNICAMP) Campinas Brazil

**Keywords:** balancing selection, chromosomal rearrangements, epistasis, linkage disequilibrium

## Abstract

Natural selection is known to favor specific gene combinations, thereby shaping the evolution of recombination rates, often through epistatic interactions. However, the dynamics of these interacting factors within natural populations remain poorly understood. In this study, we investigate the long‐term maintenance of a complex polymorphism involving linked, nonoverlapping chromosomal inversions in a natural population of *Drosophila mediopunctata*. Remarkably, even after 30 years—equivalent to roughly 340 generations—two major features have remained unexpectedly stable: the linkage disequilibrium (LD) between inversions, which deviates significantly from the theoretical prediction of decay, and a consistent seasonal cycle pattern of heterozygous excess and homozygous deficiencies. We explored the roles of recombination suppression, epistatic selection, and overdominance in maintaining this stability, examining their alignment with previously described patterns. Our findings reveal that moderate selection coefficients, such as *s* = 0.0407, are sufficient to maintain the observed LD for the most common haplotypes, albeit leading to an unstable equilibrium. Simulations further reveal that the introduction of overdominance stabilizes the system, enabling the long‐term persistence of this complex inversion polymorphism across various frequency scenarios. The stability of this system appears to hinge on a delicate balance between LD, recombination rates, and selective pressures, with overdominance playing a critical role. Our findings highlight the significance of epistatic interactions and selective pressures in shaping evolutionary pathways in natural populations and offer a compelling example of natural selection acting on a complex inversion polymorphism, providing valuable insights into the evolutionary dynamics governing inversion systems.

## Graphical abstract

It has become increasingly clear that natural selection favors haplotypes that draw functionally related genes together, often targeting mechanisms that control recombination rates. In this paper, we show that the long‐term maintenance of linkage disequilibrium between linked non‐overlapping chromosomal inversions in a Drosophila species may arise from a combination of reduced recombination, epistatic selection and balancing selection.

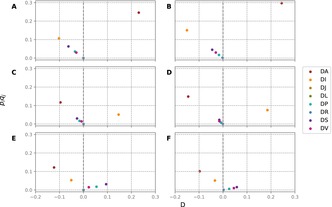

## 1 Introduction

Recombination rates are a target of natural selection, as are the alleles whose combination they modulate (e.g., Levitan 1961; Ortiz‐Barrientos, Engelstädter, and Rieseberg 2016; Winbush and Singh 2022). Pál and Hurst (2003) demonstrated that functionally related genes tend to cluster within eukaryotic genomes, with recombination rates correlating with gene dispensability. This correlation suggests that haplotypes that gather alleles of functionally related genes are often favored by selection, supporting the hypothesis that these genes interact through positive epistasis (Ortiz‐Barrientos, Engelstädter, and Rieseberg 2016). In the presence of positive epistasis, natural selection favors mechanisms that preserve advantageous allelic combinations, leading to strong linkage disequilibrium (LD).

Several authors have used two‐locus, two‐allele models to analytically explore the conditions for equilibrium under epistatic selection, which can provide a clearer understanding of how recombination rates are shaped by selective pressures (e.g., Lewontin and Kojima 1960; Hastings 1981; Asmussen and Clegg 1982; Pontz, Hofbauer, and Bürger 2018). These models consistently highlight that maintaining polymorphisms at each locus imposes stringent conditions, often requiring overdominance (Pontz, Hofbauer, and Bürger 2018) and involving a delicate balance between recombination rates, LD, and selection intensity (e.g., Lewontin and Kojima 1960; Hastings 1981). In this context, the recombination rate in an epistatic system is often constrained by the strength of selection, with mechanisms that promote tight linkage and reduce recombination being selectively favored.

Recombination rates can be altered by mechanisms that change the crossover rate between loci or by mechanisms that alter their linkage relationships (i.e., the physical location of one locus relative to another) or both (Ortiz‐Barrientos, Engelstädter, and Rieseberg 2016). Among the latter, chromosomal rearrangements, such as paracentric inversions, play a clear role in modulating recombination (Stapley et al. 2017). Paracentric chromosomal inversions suppress recombination in heterokaryotypes (Sturtevant 1926), and both theory and empirical studies suggest they can significantly impact overall genetic variation. For instance, inversions can preserve beneficial combinations of genetic variants (Fuller et al. 2016), alter the expression of genes near inversion breakpoints (Puig, Cáceres, and Ruiz 2004), and even act as potential modifiers of clinical phenotypes (Nomura et al. 2018). Moreover, inversions can generate complex patterns of gene flow by affecting crossover rates, potentially along entire chromosomes (Navarro et al. 1997; Crown et al. 2018). At the population level, inversions can dramatically reduce the number of recombinant individuals. Moreover, substantial evidence shows that increased LD due to inversions may play a role in speciation events (Ortiz‐Barrientos, Engelstädter, and Rieseberg 2016; White, Snook, and Eyres 2020).

In many *Drosophila* species, recombination inhibition mediated by inversions seems essential for maintaining the integrity of adaptive gene complexes (Dobzhansky 1947; Ananina et al. 2004; Anderson et al. 2005; Kennington, Partridge, and Hoffmann 2006; Joron et al. 2011; see Singh 2008 for a review). Furthermore, Wellenreuther and Bernatchez (2018) showed that balancing selection often plays a role in maintaining inversion polymorphisms.

Several *Drosophila* species exhibit a higher‐order phenomenon where multiple inversions on the same chromosome display high LD, even when they do not overlap. This suggests that the conditions for internal equilibrium, where complex polymorphisms are kept with LD, might be less restrictive than those predicted by analytical approaches, at least in the *Drosophila* genus (cf. Levitan and Salzano 1959; Singh 2008; Fuller et al. 2020; Navarro‐Dominguez et al. 2022). For instance, in *Drosophila subobscura*, recombination is nearly entirely inhibited in heterokaryotypes both inside and outside the inverted segment of the O chromosome (Pegueroles et al. 2010). As a result, recombinant individuals are extremely rare in natural populations (Krimbas and Zouros 1969; Fontdevila et al. 1983). Despite many examples, few have undergone long‐term studies that thoroughly assess the populational parameters involved in the underlying selection hypotheses. One exception is *D. robusta*: inversions on opposite arms of the X chromosome exhibit strong linkage, and inversion haplotypes show an intricate pattern of geographic and temporal variation, in a rather dynamic system shaped by inversion polymorphism and LD (Carson 1958; Levitan 1955, 1961, 1973). Recent studies from the early 2000s have provided strong evidence of the influence of climate change on this system, with a parallel increase in the frequency of southern‐associated arrangements in different populations (Levitan and Etges 2005; Etges, Arbuckle, and Levitan 2006).

These findings are further exemplified by the Neotropical species *Drosophila mediopunctata*, which offers additional insights into the role of chromosomal inversions in shaping complex polymorphisms. *D. mediopunctata* has six pairs of chromosomes: five pairs of rods and one pair of dots. A total of 23 inversions have been described in chromosomes IV, II, and X (Klaczko 2006). Some have been associated with different phenotypic effects, such as the “sex‐ratio” trait associated with chromosome X karyotype (Carvalho, Peixoto, and Klaczko 1989), and significant associations with wing size and abdominal pigmentation with chromosome II inversions (Hatadani et al. 2004; Hatadani and Klaczko 2008). Chromosome II is highly polymorphic for paracentric inversions, which can be grouped into two regions: there are nine inversions in the proximal region (the most frequent being PA0, PB0, and PC0) and eight in the distal (the most frequent being DA, DI, DS, DV, and DP) (Ananina et al. 2002). Inversions of each region overlap so that heterozygous form large inversion loops which effectively span the entire region (see Ananina et al. 2002, Figure 1).

**FIGURE 1:**
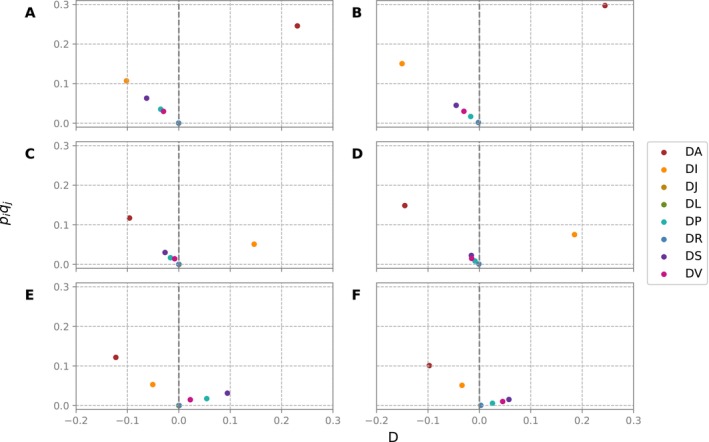
Linkage disequilibrium patterns for haplotypes associated with the proximal inversions PA0, PB0, and PC0. The left panels (A, C, E) show the patterns observed in the late 1980s (data from Peixoto and Klaczko 1991), while the right panels (B, D, F) display the patterns observed in 2015–2016. The first row (A, B) corresponds to haplotypes associated with the PA0 inversion, the second row (C, D) with the PB0 inversion, and the third row (E, F) with the PC0 inversion.

While *D. mediopunctata* males lack recombination (Cavasini, Carvalho, and Klaczko 2010), recombination between non‐overlapping inversions of the distal and proximal regions does occur in double heterozygous females, though at a significantly reduced rate (*r* = 0.3% with a 95% CI of 0.1%–0.8%). This reduction is not observed in double homozygotes (~50%), while single homozygotes show intermediate recombination rates (~18%) (Hatadani and Klaczko 2018). These findings are consistent with the crossover interference hypothesis (Gong, McKim, and Hawley 2005; Koury 2023) but could also be explained by some form of karyotype‐dependent genic modifier of recombination (Chinnici 1971; Ortiz‐Barrientos, Engelstädter, and Rieseberg 2016; Winbush and Singh 2022; Rybnikov et al. 2023).

The first major investigation of the populational dynamics of these inversions analyzed data from collections carried out between 1986 and 1988 at the Parque Nacional do Itatiaia (Rio de Janeiro State, Brazil) by L. B. Klaczko and collaborators. Peixoto and Klaczko (1991) examined 1065 males from eight of these collections. Their analysis revealed strong LD between distal and proximal chromosomal arrangements, with a standardized LD (*D*′) close to 1 for the most frequent haplotypes. Using the Linkage Disequilibrium Analysis method (see Thomson and Klitz 1987), they identified patterns of selection for the haplotypes DA‐PA0, DI‐PB0, and three distal inversions that positively associated with PC0: DS, DP, and DV. Klaczko, Otto, and Peixoto (1990) analyzed inversion karyotype data and found a consistent excess of heterozygotes for proximal inversions, which, given the LD with distal inversions, likely indicates an excess of double heterokaryotypes. Finally, Ananina et al. (2004—see also Klaczko 2006) described a seasonal pattern in haplotype frequency variation in this population, where the frequency of the DA‐PA0 haplotype increased during colder months and decreased in warmer months, alternating with haplotypes sharing the PC0 proximal inversion (DS, DP, and DV).

In this study, we re‐examine this complex inversion polymorphism at the Parque Nacional do Itatiaia by analyzing flies collected nearly 30 years after the initial collections made in the 1980s. We replicate the sampling design and analysis of the chromosome II inversions, evaluating whether the patterns we found are consistent with the hypotheses proposed in previous studies. We extend the analysis by exploring possible fitness scenarios and population dynamics that might provide insights into the processes governing this intriguing system.

## 2 Materials and Methods

### 2.1 Fly Samples and Inversion Frequencies Estimation

We collected *D. mediopunctata* from the Parque Nacional do Itatiaia on five different occasions: three in 2015 and two in 2016, covering all seasons (Table 1). The three collection sites—which included points at altitudes varying from 750 m to 1070 m—matched the collection points used in the late 80's. A similar approach was employed by Etges, Arbuckle, and Levitan (2006) when resurveying the chromosomal polymorphism of *D. robusta*. To estimate inversion frequencies, we analyzed 145 males using the male sample method (Arnold 1981; Klaczko 1995). Namely, we crossed every single male with up to three virgin females from the ITC29I strain, which is homokaryotypic for the DI‐PB0 haplotype in chromosome II. We then cytologically examined up to eight larvae from each cross, which allows for the identification of the male genotype with an error rate of < 1%.

**TABLE 1:** Sample collection dates and locations (altitude in meters) at the Parque Nacional do Itatiaia (Rio de Janeiro—Brazil).

Seasonal stage	Year	Month	Days	Altitude (m)
Late summer	2015	March	17–20	750; 1070
Late winter	2015	September	19–22	750; 950; 1070
Mid spring	2015	November	14–17	750; 950; 1070
Late summer	2016	March	17–19	750; 950; 1070
Late Fall	2016	June	13–16	750; 950; 1070

### 2.2 Disequilibrium Pattern Analysis

We applied the exploratory analysis developed by Thomson and Klitz (1987) to our data to assess how the LD patterns evolved in this population after nearly 30 years. To avoid sampling effects, we analyzed the disequilibrium patterns only for haplotypes with frequencies above 5% and adopted standardized LD values for all the comparative analyses. We produced three sets of graphs, one for each set of haplotypes sharing the proximal inversions PA0, PB0, and PC0.

### 2.3 Notation and Conventions

For simplicity, we treated overlapping inversions (e.g., DA and DI) as alleles of the same locus, and nonoverlapping inversions (e.g., DA and PA0) as alleles of different loci, following Peixoto and Klaczko (1991). Letters *A* and *B* represent different loci. *A* (uppercase) denotes the allele of interest at locus *A*, and *a* (lowercase) represents other alleles at the same locus. Similarly, *B* represents the allele of interest at locus *B*, and *b* represents other alleles at that locus. A given haplotype of interest (e.g., DA‐PA0 or DI‐PB0) is referred to as *AB*, while recombinant haplotypes involving one of the inversions that form this haplotype are referred to as *Ab* and *aB*. Any haplotype with inversions different from the one being analyzed is referred to as *ab*.

Allelic frequencies are represented by *p*: *p*_A_ represents the frequency of allele *A*, and (1−*p*_A_) the frequency of all remaining alleles at locus *A*; *p*_B_ stands for the frequency of allele *B*, and (1−*p*_B_) the frequency of all remaining alleles at locus B. The frequencies of haplotypes (or gametic types) are denoted by *g*, with varying subscripts according to each application. The frequency of karyotypes (combinations of haplotypes) is given by *Z*.

Subscripts of *Z* and *g* change depending on the context: when dealing with the same system of alleles of interest outlined above, *g*_*AB*_, *g*_*Ab*_, *g*_*aB*_, and *g*_*ab*_ give the frequencies of haplotypes *AB*, *Ab*, *Ab*, and *ab*, respectively, while *Z*_*AB/AB*_ denotes the frequency of the homokaryotype *AB*/*AB*, for example. Other subscripts are used when grouping these haplotypes (e.g., *R* stands for recombinant haplotypes *Ab* and *aB*) or when dealing with combinations of every haplotype observed (*u* for any haplotype; *v* for any other haplotype, and *w* for any third haplotype that is neither *u* nor *v*).

### 2.4 Linkage Disequilibrium

We used the chi‐square statistic to test for non‐random association between distal and proximal arrangements, following Weir's method (Weir 1996):

(1) $$X_{\mathrm{AB}}^{2}={\left[\frac{{\hat{D}}_{\mathrm{AB}}-\varepsilon D_{0}}{\sqrt{\mathrm{Var}\left(D_{0}\right)}}\right]}^{2}$$

(2) $$\varepsilon D_{0}=\frac{2n-1}{2n}D_{0}$$

(3) $$\mathrm{Var}\left(D_{0}\right)=\frac{1}{2n}{\tilde{p}}_{A}\left(1-{\tilde{p}}_{A}\right){\tilde{p}}_{B}\left(1-{\tilde{p}}_{B}\right)+\left(1-2p_{A}\right)\left(1-2p_{B}\right)D_{0}-{D_{0}}^{2}$$

where ${\hat{D}}_{\mathrm{AB}}$ is the observed LD between alleles *A* and *B*, n is the sample size (number of individuals analyzed), and $D_{0}$ is D under the null hypothesis.

With $D_{0}=0$, equation (1) simplifies to:

(4) $$X_{\mathrm{AB}}^{2}=\frac{2n{\hat{D}}_{\mathrm{AB}}^{2}}{{\tilde{p}}_{A}\left(1-{\tilde{p}}_{A}\right){\tilde{p}}_{B}\left(1-{\tilde{p}}_{B}\right)}$$

After attributing *p* values for each haplotype, we verified significance after applying Benjamini‐Hochberg procedure for multiple testing.

### 2.5 Hardy–Weinberg Equilibrium

We assessed whether inversion haplotypes (i.e., each given combination of distal and proximal inversions) randomly associate with form karyotypes in this population, by testing for Hardy–Weinberg equilibrium (HWE). We calculated a *D* value for each inversion karyotype, where *D* is the difference between the observed and expected inversion karyotype frequencies under HWE (Weir 1996):

(5) $$Z_{\mathrm{uu}}=g_{u}^{2}+D_{\mathrm{uu}}$$

(6) $$Z_{\mathrm{uv}}=2g_{u}g_{v}-2D_{\mathrm{uv}},\;v\neq u$$

where *u* stands for any haplotype and *v* stands for any other haplotype. Hence, $g_{u}$ is the frequency of any haplotype, while $g_{v}$ is the frequency of any other haplotype in the population; $Z_{\mathrm{uu}}$ is the frequency of the homokaryotype while $Z_{\mathrm{uv}}$ is the frequency of each heterokaryotype.

We then applied Fisher's variance approximation to compute the variance of the maximum likelihood estimation (MLE) for homozygous – $\;\mathrm{Var}\left({\hat{D}}_{\mathrm{uu}}\right)$ – and heterozygous – $\mathrm{Var}\left({\hat{D}}_{\mathrm{uv}}\right)$ – individuals.

(7) $$\mathrm{Var}\left({\hat{D}}_{\mathrm{uu}}\right)=\frac{1}{n}\left[g_{u}^{2}{\left(1-g_{u}\right)}^{2}+{\left(1-2g_{u}\right)}^{2}\;D_{\mathrm{uu}}-D_{\mathrm{uu}}^{2}\right]$$

(8) $$\mathrm{Var}\left({\hat{D}}_{\mathrm{uv}}\right)=\frac{1}{2n}\;\left\{g_{u}g_{v}\;\left[\left(1-g_{u}\right)\left(1-g_{v}\right)+g_{u}g_{v}\right]-\left[{\left(1-g_{u}-g_{v}\right)}^{2}-2{\left(g_{u}-g_{v}\right)}^{2}\right]D_{\mathrm{uv}}+\sum_{w\neq u,v}\left(g_{u}^{2}\;D_{\mathrm{vw}}+g_{u}^{2}D_{\mathrm{uw}}\right)-{2D}_{\mathrm{uv}}^{2}\right\}$$

where *w* is any other allele that is neither *u* nor *v*.

Given that for large samples MLE $\hat{D}$ is approximately normally distributed under $H_{0}$, a standard normal variate (*z*) can be constructed as

(9) $$z=\frac{\hat{D}}{\sqrt{\mathrm{Var}\left(\hat{D}\right)}}$$

We used these formulae (from Weir 1996) to test each inversion karyotype (with expected number of individuals above five) for HWE, attributing significance after applying Benjamini‐Hochberg procedure for multiple testing.

### 2.6 Comparison With Karyotype Frequencies of Klaczko, Otto, and Peixoto (1990)

To compare our data with Klaczko, Otto, and Peixoto (1990), we reclassified it based on the proximal inversion, reducing the number of karyotypes to six: three homokaryotypes (PA/PA; PB/PB; PC/PC) and three heterokaryotypes (PA/PB; PA/PC and PB/PC). We then analyzed our results and those of Klaczko, Otto, and Peixoto (1990) as follows: first, we calculated *D* and *z* for each karyotype from flies collected on each occasion separately. Then, to take into account the different moments of the seasonal cycle described by Ananina et al. (2004), we did the same calculations on samples collected just after the warm season [March for our data, February for Klaczko, Otto, and Peixoto 1990s] and just after the cold season [September for both datasets].

### 2.7 Linkage Disequilibrium Decay

Given the incomplete recombination arrest in double heterozygote females, one would expect the LD to have decreased over generations due to recurrent recombination unless counteracting forces are at play. After ruling out the possibility that the low recombination rate estimated from morphological markers could result from high mortality of recombinants at early developmental stages (Appendix, Section 1 in File S2), we used the recombination rate (*r =* 0.3%) estimated for double heterokaryotype females by Hatadani and Klaczko (2018), adjusting for the fact that males do not recombine $\left(r/2\right)$. We estimated expected the expected LD values under a neutral model, following Lewontin and Kojima (1960):

(10) $$D_{t}={\left(1-r\right)}^{t}D_{0}$$

To estimate the number of generations (*t*) between the two datasets (1986/88 and 2015/2016), we used the average temperature in Itatiaia over the past 30 years (18.8°C), the corresponding development time of *D. mediopunctata* at that temperature, which is approximately 4.5 weeks per generation (see details in Appendix, Section 2 in File S2). Assuming a discrete generation model and a 360‐day year (for ease of calculation), we estimated that t≈339 for a 30‐year period. We then calculated $D_{t}$ and tested whether the current D values significantly deviate from the expected LD, assuming decay under a neutral model. This was done using Equation 10, with $D_{t}$ as D under the null hypothesis $\left(D_{0}\right)$.

### 2.8 Selection Coefficients Estimation

Assuming that LD remained stable in the Itatiaia population between the collections made in 1986 and in 2016 and considering the expected decay of LD under neutral conditions, we sought to estimate the magnitude of selection necessary to counterbalance the tendency toward linkage equilibrium. We focused on the observed LD between inversions DA and PA0, given their frequencies fluctuate around 0.5 in this population, allowing us to approximate $g_{\mathrm{AB}}\approx g_{\mathrm{ab}}$. We used the same recombination rate (*r* = 0.0015) as in the LD decay analysis. We assumed no genotypic effect on fitness, meaning both non‐recombinant (*NR*) haplotypes are equally beneficial, while recombinant (*R*) haplotypes are equally detrimental in any genotype. We denoted the frequency of non‐recombinant haplotypes ($g_{\mathrm{AB}}+g_{\mathrm{ab}}$) as $g_{\mathrm{NR}}$, and the frequency of recombinant haplotypes ($g_{\mathrm{aB}}+g_{\mathrm{Ab}}$) as $g_{R}$. The equilibrium frequency of recombinant haplotypes (${\hat{g}}_{R}$) was estimated as the average frequency of recombinant haplotypes (i.e., combinations of DA or PA0 that were not DA‐PA0) observed in the Itatiaia population between 1986 (Peixoto and Klaczko 1991) and 2016.

The frequencies of each genotype are, respectively:

$$Z_{\mathrm{NR}/\mathrm{NR}}=Z_{\mathrm{AB}/\mathrm{AB}};Z_{\mathrm{AB}/\mathrm{ab}};Z_{\mathrm{ab}/\mathrm{ab}}$$

$$Z_{\mathrm{NR}/R}=Z_{\mathrm{AB}/\mathrm{Ab}};Z_{\mathrm{AB}/\mathrm{aB}};Z_{\mathrm{ab}/\mathrm{Ab}};Z_{\mathrm{ab}/\mathrm{aB}}$$

$$Z_{R/R}=Z_{\mathrm{Ab}/\mathrm{Ab}};Z_{\mathrm{aB}/\mathrm{aB}};Z_{\mathrm{Ab}/\mathrm{aB}}$$

and the resulting fitness values for these groups are as follows:

**Table:** 

two non‐recombinant chromosomes	$Z_{\mathrm{NR}/\mathrm{NR}}$	$w_{\mathrm{NR}/\mathrm{NR}}=1$
one non‐recombinant and one recombinant chromosome	$Z_{\mathrm{NR}/R}$	$w_{\mathrm{NR}/R}=1-\mathrm{hs}$
two recombinant chromosomes	$Z_{R/R}$	$w_{R/R}=1-s$

We therefore account for the presence of position effects, i.e., $w_{\mathrm{AB}/\mathrm{ab}}\neq w_{\mathrm{Ab}/\mathrm{aB}}$. However, since the frequency of double heterokaryotype resulting from the combination of recombinant chromosomes is negligible due to their extremely low occurrence in the population, this assumption is not expected to cause significant deviations from models that assume $w_{\mathrm{AB}/\mathrm{ab}}=w_{\mathrm{Ab}/\mathrm{aB}}$.

This assumption allows the frequency change due to selection to be described as (Gillespie 2010):

(11) $$∆{g_{\mathrm{NR}}}_{s}=\frac{g_{\mathrm{NR}}g_{R}s\;\left[g_{\mathrm{NR}}h+g_{R}\left(1-h\right)\right]}{1-2g_{\mathrm{NR}}g_{R}\mathrm{hs}-{g_{R}}^{2}s}$$

The loss of non‐recombinant haplotypes will be governed by the rate of recombination in double heterozygous, given by

(12) $$∆{g_{\mathrm{NR}}}_{r}=-2g_{\mathrm{AB}}g_{\mathrm{ab}}r+2g_{\mathrm{Ab}}g_{\mathrm{aB}}r$$

For extremely low recombination rates and recombinant haplotype frequencies, $2g_{\mathrm{Ab}}g_{\mathrm{aB}}r\approx 0$. Assuming $g_{\mathrm{AB}}=g_{\mathrm{ab}}$, we can express $g_{\mathrm{NR}}=2g_{\mathrm{AB}}$. A simple measure of the selection coefficient (*s*) necessary to achieve the equilibrium can thus be calculated as:

$$∆{{\hat{g}}_{\mathrm{NR}}}_{r}+∆{{\hat{g}}_{\mathrm{NR}}}_{S}=0$$

(13) $$2{\hat{g}}_{\mathrm{AB}}{\hat{g}}_{\mathrm{ab}}r=\frac{{\hat{g}}_{\mathrm{NR}}{\hat{g}}_{R}s\;\left[{\hat{g}}_{\mathrm{NR}}h+{\hat{g}}_{R}\left(1-h\right)\right]}{1-2{\hat{g}}_{\mathrm{NR}}{\hat{g}}_{R}\mathrm{hs}-{\hat{g}}_{R}^{2}s}$$

given that *r* and *h* are known and $g_{\mathrm{AB}}g_{\mathrm{ab}}={\left(\left(1-{\hat{g}}_{R}\right)/2\right)}^{2}$, we can calculate *s* as follows:

For *h* = 0:

(14) $$s=\frac{g_{\mathrm{NR}}r}{2-4g_{\mathrm{NR}}+2g_{\mathrm{NR}}^{2}-g_{\mathrm{NR}}r+2g_{\mathrm{NR}}^{2}r-g_{\mathrm{NR}}^{3}r}$$

For *h* > 0:

(15) $$s=\frac{\frac{g_{\mathrm{NR}}^{4}r}{2}+g_{\mathrm{NR}}^{3}rg_{R}-\frac{g_{\mathrm{NR}}^{2}g_{R}^{2}r}{2}}{g_{\mathrm{NR}}g_{R}\left(g_{\mathrm{NR}}h+g_{R}\left(1-h\right)\right)-g_{\mathrm{NR}}^{3}g_{R}\mathrm{rh}+\frac{g_{\mathrm{NR}}^{2}g_{R}^{2}r}{2}}$$

We calculated the selection coefficient under the scenarios of complete dominance (*h* = 0 or *h* = 1) and no dominance (*h* = 0.5).

### 2.9 Epistatic Model

We conducted simulations under the assumption of no dominance to explore the dynamics of the fitness scheme outlined above. For this purpose, we developed the Epistatic Selection Simulator (File S1), a Python‐based tool designed to simulate population dynamics under different epistatic selection models. The simulator is based on a simple two‐locus, two‐allele model with random mating and discrete generations, where alleles may be either non‐recombinant (*NR*) or recombinant (*R*). We ignored the effects of mutation, gene conversion, and genetic drift, assuming that selection acts solely through viability differences. Additionally, we applied a single recombination rate for double (i.e., $w_{\mathrm{AB}/\mathrm{ab}}$) and single heterozygous (i.e., $w_{\mathrm{AB}/\mathrm{Ab}}$) and assumed the frequency of recombinant haplotypes has remained constant over the past three decades.

Let *A* and *a* be the alleles of *locus A*, and *B* and *b* be the alleles of *locus B*. Haplotype frequencies are denoted by $g_{\mathrm{AB}},g_{\mathrm{ab}},g_{\mathrm{Ab}},g_{\mathrm{aB}}$. Here, $g_{\mathrm{AB}}$ and $g_{\mathrm{ab}}$ represent the frequency of non‐recombinant haplotypes (*NR*), while $g_{\mathrm{Ab}}$ and $g_{\mathrm{aB}}$ represent the frequency of recombinant haplotypes (*R*). We use the classical definition of LD coefficient (*D*), in which *D* quantifies the deviation from expected haplotype frequencies under equilibrium. The frequency of each haplotype can thus be calculated as:

$$g_{\mathrm{AB}}=p_{A}p_{B}+D_{\mathrm{AB}}$$

$$g_{\mathrm{ab}}=\left(1-p_{A}\right)\left(1-p_{B}\right)+D_{\mathrm{ab}}$$

$$g_{\mathrm{Ab}}=p_{A}\left(1-p_{B}\right)-D_{\mathrm{Ab}}$$

$$g_{\mathrm{aB}}=\left(1-p_{A}\right)p_{B}-D_{\mathrm{aB}}$$

The fitness values for each genotype are given by the following matrix:

**Table:** 

	AB	ab	Ab	aB
AB	$w_{\mathrm{NR}/\mathrm{NR}}$	$w_{\mathrm{NR}/\mathrm{NR}}$	$w_{\mathrm{NR}/R}$	$w_{\mathrm{NR}/R}$
ab	$w_{\mathrm{NR}/\mathrm{NR}}$	$w_{\mathrm{NR}/\mathrm{NR}}$	$w_{\mathrm{NR}/R}$	$w_{\mathrm{NR}/R}$
Ab	$w_{\mathrm{NR}/R}$	$w_{\mathrm{NR}/R}$	$w_{R/R}$	$w_{R/R}$
aB	$w_{\mathrm{NR}/R}$	$w_{\mathrm{NR}/R}$	$w_{R/R}$	$w_{R/R}$

where

$$w_{\mathrm{AB}/\mathrm{AB}}=w_{\mathrm{ab}/\mathrm{ab}}=w_{\mathrm{NR}/\mathrm{NR}}=1$$

$$w_{\mathrm{AB}/\mathrm{Ab}}=w_{\mathrm{AB}/\mathrm{aB}}=w_{\mathrm{ab}/\mathrm{Ab}}=w_{\mathrm{ab}/\mathrm{aB}}=w_{\mathrm{NR}/R}=1-\mathrm{hs}$$

$$w_{\mathrm{Ab}/\mathrm{Ab}}=w_{\mathrm{Ab}/\mathrm{aB}}=w_{\mathrm{aB}/\mathrm{Ab}}=w_{\mathrm{aB}/\mathrm{aB}}=w_{R/R}=1-s$$

### 2.10 Epistatic Model With Overdominance

We explored a second epistatic scenario that incorporates a genotypic effect on fitness. Like the first model, this one considers a two‐locus, two‐allele system; however, while the first model focuses on the impact of the number of recombinant chromosomes on fitness, the second model builds upon this by also incorporating fitness reductions associated with each homozygous *locus*. The selection coefficients $s_{r}$ and $s_{\mathrm{ho}}$ represent these effects, where $s_{r}$ denotes the fitness reduction due to each recombinant chromosome and $s_{\mathrm{ho}}$ represents the fitness decrease from each homozygous *locus*, as specified by the following matrix:

**Table:** 

	AB	ab	Ab	aB
AB	$w_{3}$	$w_{1}$	$w_{2}$	$w_{2}$
ab	$w_{1}$	$w_{3}$	$w_{2}$	$w_{2}$
Ab	$w_{2}$	$w_{2}$	$w_{4}$	$w_{5}$
aB	$w_{2}$	$w_{2}$	$w_{5}$	$w_{4}$

**Table:** 

double *NR*, double heterozygous	$w_{\mathrm{AB}/\mathrm{ab}}=w_{1}=1$
*NR/R*, homozygous/heterozygous	$w_{\mathrm{AB}/\mathrm{Ab}}=w_{\mathrm{AB}/\mathrm{aB}}=w_{\mathrm{ab}/\mathrm{Ab}}=w_{\mathrm{ab}/\mathrm{aB}}=w_{2}=1-\left(s_{r}+s_{\mathrm{ho}}\right)$
double *NR*, double homozygous	$w_{\mathrm{AB}/\mathrm{AB}}=w_{\mathrm{ab}/\mathrm{ab}}=w_{3}=1-\left(2s_{\mathrm{ho}}\right)$
double *R*, double homozygous	$w_{\mathrm{Ab}/\mathrm{Ab}}=w_{\mathrm{aB}/\mathrm{aB}}=w_{4}=1-\left(2s_{r}+2s_{\mathrm{ho}}\right)$
double *R*, double heterozygous	$w_{\mathrm{Ab}/\mathrm{aB}}=w_{4}=1-\left(2s_{r}\right)$

where

We explored the properties of the model by varying the values of $s_{r}$ and $s_{\mathrm{ho}}$ and evaluated their impact on the population dynamics.

## 3 Results

### 3.1 Linkage Disequilibrium Pattern Analysis

Thomson and Klitz (1987) showed that LD created by past or recurrent selective events often retains a distinct signature, even after several generations of random mating. This signature almost invariably differs from the patterns generated by neutral forces. Our data, analyzed using the disequilibrium pattern analysis method (Thomson and Klitz 1987), revealed LD patterns that strongly suggest selection, consistent with those described by Peixoto and Klaczko (1991). Specifically, for the proximal inversion PA0, haplotype DA‐PA0 was isolated in the positive disequilibrium space, while related haplotypes (DI‐PA0, DS‐PA0, DP‐PA0, DV‐PA0, and DR‐PA0) were distributed within the negative disequilibrium space. Similarly, for the proximal inversion PB0 selection favored the haplotype DI‐PB0. The proximal inversion PC0 had a more complex pattern, with haplotypes DS‐PC0, DV‐PC0, and DP‐PC0 on the positive disequilibrium space (see Figure 1). It is worth noting that DV seems to have replaced DP as the second most common haplotype involving PC0. These presumably selected haplotypes exhibited high standardized LD coefficients (see Table 2 and Figure S1).

**TABLE 2:** Haplotype frequencies and linkage disequilibrium observed in the Itatiaia population in 2015 and 2016.

Haplotype	*n*	Frequency (*g*)	Cumulative *g*	*D*	*D*′	*χ* ^2^
DA‐PA0	155	0.5345	0.534	0.2433	1	278.1716
DI‐PB0	76	0.2621	0.797	0.1831	0.93	238.2768
DS‐PC0	21	0.0724	0.869	0.057	0.8464	81.921
DV‐PC0	15	0.0517	0.921	0.0421	1	69.1313
DP‐PC0	9	0.0310	0.952	0.0253	1	40.5931
DI‐PC0	7	0.0241	0.976	−0.0292	−0.5471	7.9631
DA‐PB0	2	0.0069	0.983	−0.1434	−0.9541	120.3794
DS‐PB0	2	0.0069	0.990	−0.0159	−0.6979	282.2389
DA‐PC0	1	0.0034	0.993	−0.098	−0.966	74.1185
DS‐PC1	1	0.0034	0.997	0.0032	1	11.1217
DR‐PC0	1	0.0034	1.000	0.0028	1	4.3855
Total	290					

*Note: n* stands for the total number of observed chromosomes. *D* stands for the linkage disequilibrium, whereas *D*′ stands for the standardized disequilibrium values of each haplotype. The last column shows a chi‐square statistic for the hypothesis of linkage equilibrium *H*_0_: DAB = 0, as described in Weir (1996). All values are significant at α = 0.05 after correction for multiple tests by Benjamini‐Hochberg procedure.

### 3.2 Haplotype Frequencies and Linkage Disequilibrium

The five most common haplotypes (DA‐PA0, DI‐PB0, DS‐PC0, DV‐PC0, and DP‐PC0) accounted for 95.17% of the segregating haplotypes in a very polymorphic population (see Table 2). Accordingly, there was strong and statistically significant LD between distal and proximal inversions for most inversion combinations, with levels remarkably similar to those previously estimated by Peixoto and Klaczko (1991). This suggests that the previously observed deviation from random combinations between distal and proximal inversions has persisted in this population over the past 30 years.

### 3.3 Genotype Frequency Deviations

We also investigated whether haplotypes of homologous chromosomes associate randomly during karyotype formation. We tested the null hypothesis that the population exhibits HWE proportions ($H_{0}:D_{\mathrm{AB}}=0$) against the alternative hypothesis of homokaryotype deficiency and double heterokaryotype excess $\left(H_{1}:D_{\mathrm{AB}}<0\right)$ using a one‐tailed *z* score. The results revealed an overall nominal deficiency of homokaryotypes and a corresponding excess of heterokaryotypes, with a few exceptions. Specifically, we observed a deficiency of homozygotes for DA‐PA0 (*z* = −2.182; *p* = 0.0145) and DI‐PB0 (*z* = −1.883; *p* = 0.0298), and an excess of the heterozygous genotype DA‐PA0/DI‐PB0 (*z* = −2.197; *p* = 0.0139). However, only the DA‐PA0/DA‐PA0 genotype showed a significant deviation from HWE after correcting for multiple tests (Table 3).

**TABLE 3:** Haplotype combination frequencies and disequilibrium coefficients (*D*) against frequencies expected by Hardy–Weinberg equilibrium for the Itatiaia population in 2015 and 2016.

Karyotype	*n*	Frequency (*Z*)	*D*	*z*	*p*
**DA‐PA0/DA‐PA0**	**35**	**0.2414**	**−0.0443**	**−2.1822**	**0.0145**
DI‐PB0/DI‐PB0	6	0.0414	−0.0273	−1.8833	0.0298
DS‐PC0/DS‐PC0	1	0.0069	0.0017		
DV‐PC0/DV‐PC0	1	0.0069	0.0042		
DA‐PA0/DA‐PB0	2	0.0138	−0.0032		
DA‐PA0/DA‐PC0	1	0.0069	−0.0016		
DA‐PA0/DI‐PB0	50	0.3448	−0.0323	−2.1977	0.0139
DA‐PA0/DI‐PC0	4	0.0276	−0.0009		
DA‐PA0/DS‐PB0	1	0.0069	0.0002		
DA‐PA0/DS‐PC0	12	0.0828	−0.0027	−0.3351	0.3688
DA‐PA0/DS‐PC1	1	0.0069	−0.0016		
DA‐PA0/DV‐PC0	7	0.0483	0.0035	0.5038	0.3072
DA‐PA0/DP‐PC0	7	0.0483	−0.0076		
DI‐PB0/DI‐PC0	1	0.0069	0.0029		
DI‐PB0/DR‐PC0	1	0.0069	−0.0025		
DI‐PB0/DS‐PC0	6	0.0414	−0.0017	−0.2457	0.4029
DI‐PB0/DV‐PC0	5	0.0345	−0.0037		
DI‐PB0/DP‐PC0	1	0.0069	0.0047		
DI‐PC0/DS‐PC0	1	0.0069	−0.0017		
DI‐PC0/DV‐PC0	1	0.0069	−0.0022		
DS‐PB0/DP‐PC0	1	0.0069	−0.0032		
Total	145				

*Note: n* stands for the total number of individuals. *D* stands for the disequilibrium coefficient of each genotype. The last two columns show a *z*‐score statistic for the hypothesis of no deviations from HWE assumptions, *H*_0_: *D* = 0, as described in Weir (1996) and the corresponding *p* value. The *z* and *p*‐values are not shown for classes with expected values below 5. Bold values are significant at α = 0.05 after Benjamini‐Hochberg procedure for multiple testing.

A previous study by Klaczko, Otto, and Peixoto (1990) reported a slight excess of heterozygous individuals across all male adult samples from eight collecting dates. In one instance, there was a significant deviation from HWE, with an excess of PC/PB [which may be interpreted as nearly synonymous with DS (or DP or DV)‐PC0/DI‐PB0 double heterokaryotypes] in the February sample. Considering the cyclic frequency variation of haplotypes DA‐PA0 (fall/winter frequency peak) and DS‐PC0 (spring/summer frequency peak) described for the 80s collections (Ananina et al. 2004), we hypothesized that mixing collections from different stages of seasonal variation could obscure meaningful signals in karyotype frequency variation.

**FIGURE 2:**
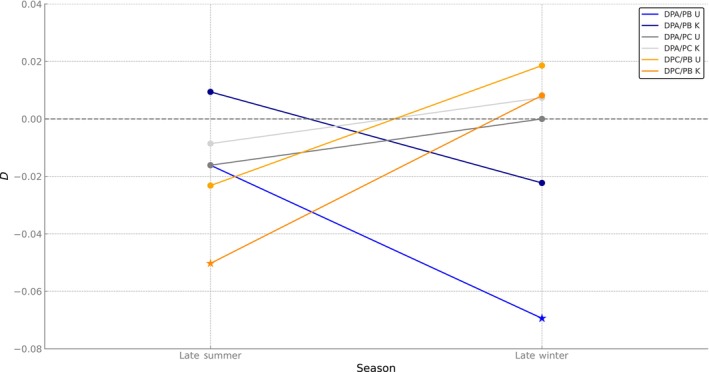
Heterokaryotype frequency deviation from Hardy–Weinberg equilibrium expectation as measured by *D* (disequilibrium coefficient) for heterokaryotypes PA/PB, PC/PB, and PA/PC for datasets from Klaczko, Otto, and Peixoto (1990) [K] and from collections carried out ~30 years later [U], separated by season. Stars indicate *D* values significantly different from zero.

To make our results comparable, we grouped karyotypes following the classification used by Klaczko, Otto, and Peixoto (1990) and analyzed each dataset separately, focusing on collections conducted just after the warm and cold seasons. Both datasets revealed a pronounced deficiency of the homokaryotype PC/PC (significant in both datasets) and an excess of the heterokaryotype PB/PC (significant in the 80s dataset) in collections made after the warm season (February and March). A similar pattern was observed in collections made after the cold season (September), but involving inversions PA and PB: both datasets showed a pronounced deficiency of the homokaryotype PA/PA (significant in the 2010 dataset) and an excess of the heterokaryotype PB/PA (significant in the 2010 dataset) (see Figure 2 and Table 4). The PB haplotype, consistently found in heterozygous excess, repeatedly exhibited a deficiency of its homozygous state (Figure S2 and Table 4).

**TABLE 4:** Haplotype frequencies and disequilibrium coefficients (*D*) compared to those expected under Hardy–Weinberg equilibrium, grouped as in Klaczko, Otto, and Peixoto (1990).

Season	Dataset	Karyotype	*n*	Frequency (*Z*)	*D*	*z*	*p*
Late summer	1980s	PA/PA	12	0.17647	0.00081	0.0401	0.4840
		**PB/PB**	**5**	**0.07353**	**−0.04087**	**−2.3424**	**0.0096**
		**PC/PC**	**0**	**0.00000**	**−0.05888**	**−5.8462**	**< 0.00001**
		PA/PB	18	0.26471	0.00941	0.6324	0.2635
		PA/PC	15	0.22059	−0.00860	−0.6745	0.2500
		**PC/PB**	**18**	**0.26471**	**−0.05028**	**−3.8180**	**< 0.0001**
	2010s	PA/PA	6	0.18750	−0.03223	−1.5732	0.0579
		**PB/PB**	**1**	**0.03125**	**−0.03931**	**−2.8352**	**0.0023**
		**PC/PC**	**1**	**0.03125**	**−0.03931**	**−2.8352**	**0.0023**
		PA/PB	9	0.28125	−0.01611	−1.1480	0.1255
		PA/PC	9	0.28125	−0.01611	−1.1480	0.1255
		PC/PB	6	0.18750	−0.02319	−1.8665	0.0310
Late winter	1980s	PA/PA	36	0.23841	−0.01491	−0.7196	0.2361
		PB/PB	9	0.05960	−0.01412	−0.8966	0.1848
		PC/PC	10	0.06623	0.01553	1.0009	0.1588
		PA/PB	48	0.31788	−0.02228	−1.4499	0.0737
		PA/PC	32	0.21192	0.00737	0.5215	0.3016
		PC/PB	16	0.10596	0.00816	0.7373	0.2304
	2010s	**PA/PA**	**9**	**0.25714**	**−0.06939**	**−3.6041**	**0.0002**
		**PB/PB**	**2**	**0.05714**	**−0.05082**	**−3.0552**	**0.0011**
		PC/PC	1	0.02857	0.01857	1.5957	0.9443
		**PA/PB**	**18**	**0.51429**	**−0.06939**	**−4.2249**	**< 0.00001**
		PA/PC	4	0.11429	0.00000	0.0000	0.5000
		PC/PB	1	0.02857	0.01857	2.5462	0.9946

*Note:* These values were calculated from samples collected in 1986/87 and 2015/16, during different phases of the seasonal cycle of inversion frequency fluctuations described by Ananina et al. (2004). *n* stands for the total number of individuals. *D* stands for the disequilibrium coefficient of each genotype. The last column shows a *z*‐score statistic for the hypothesis of no deviations from HWE assumptions, *H*_0_: *D* = 0, as described in Weir (1996). The *z* and *p*‐values are not shown for classes with expected values below 5. Bold values are significant at α = 0.05 after Benjamini‐Hochberg procedure for multiple testing.

### 3.4 Expected Linkage Disequilibrium Decay due to Recombination

**FIGURE 3:**
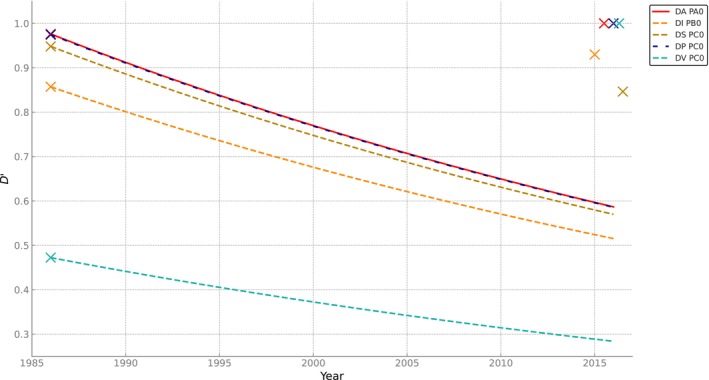
Standardized linkage disequilibrium (*D′*) levels for five inversion haplotypes of *Drosophila mediopunctata* from Parque Nacional de Itatiaia (RJ). The LD values observed in 1986 (Peixoto and Klaczko 1991) are represented by the crosses on the far left. The dashed lines show the expected LD decay under a neutral model. The crosses on the far right depict the LD values observed in 2015/2016. The 2015/2016 data points have been slightly shifted for better visualization.

Even with the reduced recombination rate estimated for double heterozygote females (*r* = 0.003), substantial LD decay would be expected over 339 generations of random mating. Under a neutral scenario, LD values are expected to drop to approximately 60.1% of their initial values. For most common haplotypes, the observed LD values differed significantly from this expectation (DA‐PA0, *p* < 0.001; DI‐PB0, *p* < 0.001; DV‐PC0, *p* < 0.001). Nevertheless, we did not find significant differences between the observed LD and the expected LD due to recombination for the haplotypes DS‐PC0 and DP‐PC0 (Table 5). This result might be related to the low frequencies of inversions DS and DP (0.083 and 0.031, respectively) and the fact that *D* is sensitive to gene frequencies (Lewontin 1964). Therefore, we adopted the statistic *D*′ for our comparisons, as it measures association independent of allele frequency. Figure 3 presents the corresponding *D*′ values for the five most frequent haplotypes in the sample.

**TABLE 5:** Current and expected linkage disequilibrium (*D*) values after decay due to recombination.

Haplotype	Observed		Expected	*χ* ^2^	*p*
	*D*_80's_	*D*_2015–2016_	*D*_339_		
**DA‐PA0**	**0.2307**	**0.2433**	**0.1295**	**82.6907**	**< 0.0001**
**DA‐PB0**	**−0.0959**	**−0.1434**	**−0.0539**	**47.8274**	**< 0.0001**
DA‐PC0	−0.1209	−0.0980	−0.0679	7.2549	0.0112
**DI‐PB0**	**0.1467**	**0.1831**	**0.0824**	**59.4105**	**< 0.0001**
DI‐PC0	−0.0507	−0.0292	−0.0285	0.0077	0.4440
DS‐PB0	−0.0268	−0.0159	−0.0150	0,0277	0.4920
DS‐PC0	0.0945	0.0570	0.0531	0.1334	0.4868
DS‐PC1	−0.0025	0.0032	−0.0014	NA	NA
DP‐PC0	0.0543	0.0253	0.0305	0.3546	0.2056
**DV‐PC0**	**0.0223**	**0.0421**	**0.0125**	**17.7757**	**< 0.0001**
DR‐PC0	0.0007	0.0028	0.0004	NA	NA

*Note: D*_80's_ stands for the linkage disequilibrium values observed in this population between 1986 and 1988. *D*_2015–2016_ stands for the linkage disequilibrium values observed in this population between 2015 and 2016. *D*_339_ stands for the expected linkage disequilibrium after 339 generations of decay due to recombination. The last two columns show a chi‐square statistic for *H*_0_ = *D*_339_. The chi‐square and *p*‐values are not shown for classes with expected values below 5. Bold values are significant at α = 0.05 after Benjamini‐Hochberg procedure for multiple testing. NA indicates $\mathrm{Var}\left(D_{0}\right)=0$.

### 3.5 Maintenance of Linkage Disequilibrium by Selection

**FIGURE 4:**
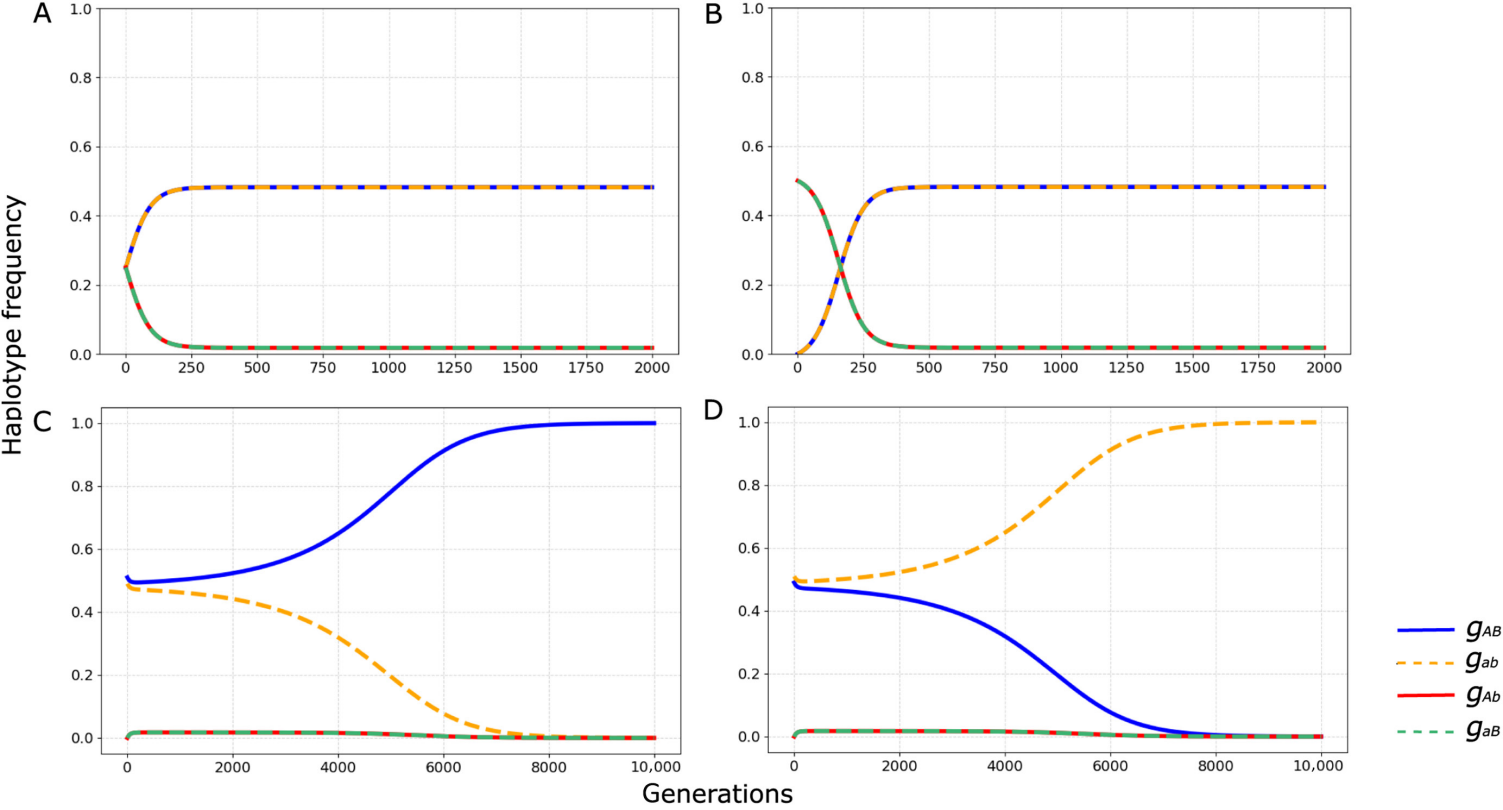
Haplotype frequency dynamics in a simple epistatic model where all recombinant haplotypes are equally disfavored (*s =* 0.041), with no dominance (*h =* 0.5) and a low recombination rate empirically estimated for double heterokaryotype females of *D. mediopunctata* (*r =* 0.0015). The upper panels (A and B) illustrate the changes in haplotype frequencies when the initial frequencies of both non‐recombinant haplotypes are identical (A—$g_{\mathrm{AB}}=g_{\mathrm{ab}}=0.25$; B—$g_{\mathrm{AB}}=g_{\mathrm{ab}}=0.0001$). The lower panels display the effects of a slight initial frequency difference between non‐recombinant haplotypes (C—$g_{\mathrm{AB}}=0.51\;\text{and}\;g_{\mathrm{ab}}=0.49$; D—$g_{\mathrm{AB}}=0.49\;\text{and}\;g_{\mathrm{ab}}=0.51$). Blue solid line: *G*_*AB*_; orange dashed line: *G*_*ab*_; green and red dashed lines: *G*_*Ab*_ and *g*_*aB*_.

**FIGURE 5:**
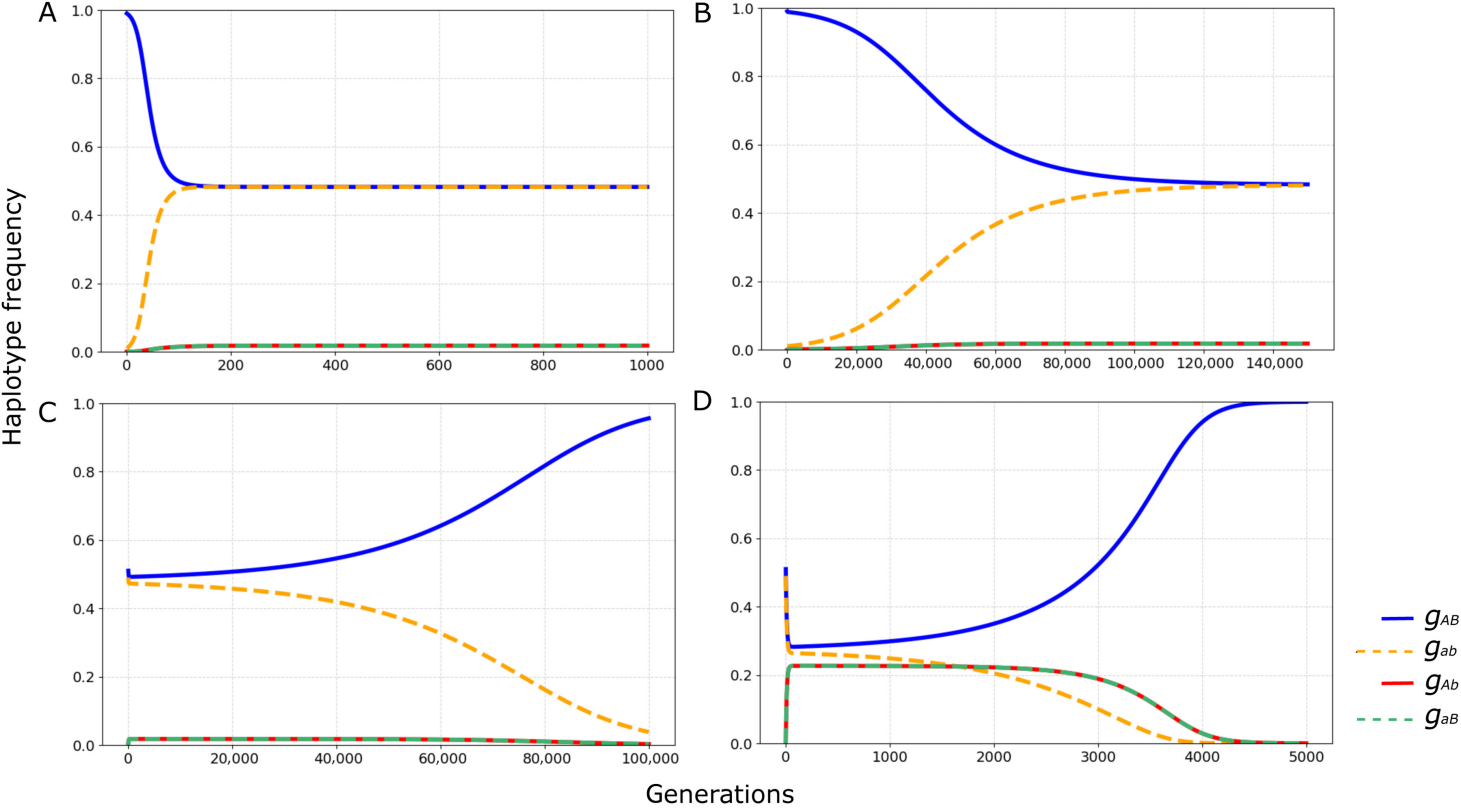
Epistatic selection with overdominance can lead to a stable equilibrium characterized by intermediate haplotype frequencies and persistence of linkage disequilibrium, depending on initial parameters. Panel A: A stable equilibrium with intermediate haplotype frequencies is achieved when there is a similar excess of double heterozygotes and recombinant haplotypes (*r* = 0.0015; initial haplotype frequencies *g*_*AB*_ = 0.99 and *g*_*ab*_ = 0.01; selection coefficient against recombinant haplotypes (*s*_*r*_) = 0.02035; and selection coefficient against homozygous loci (*s*_ho_) = 0.05). Panel B shows conditions close to the lower bound of heterozygous advantage required to maintain internal equilibrium (*r* = 0.0015; *g*_*AB*_ = 0.99 and *g*_*ab*_ = 0.01; *s*_*r*_ = 0.02035 and *s*_ho_ = 0.0008). Panel C: The loss of internal equilibrium occurs with very low heterozygous advantage (*r* = 0.0015; *g*_*AB*_ = 0.51 and *g*_*ab*_ = 0.49; *s*_*r*_ = 0.02035 and *s*_ho_ = 0.0007). Panel D: High recombination rates lead to the breakdown of internal equilibrium (*r* = 0.1; *g*_*AB*_ = 0.51 and *g*_*ab*_ = 0.49; *s*_*r*_ = 0.02035 and *s*_ho_ = 0.015). Blue solid line: *G*_*AB*_; orange dashed line: *G*_*ab*_; green and red dashed lines: *G*_*Ab*_ and *g*_*aB*_.

With no dominance (*h* = ½), we estimate that a selection coefficient of approximately 0.0407 would be sufficient to maintain the observed LD between the inversions DA and PA0 (Equation 14). For the full‐dominance epistatic model, two scenarios are possible. If the recombinant haplotype is dominant, the selection coefficient necessary to maintain a stable frequency for that haplotype is reduced to ~0.0211. Conversely, with complete dominance of the non‐recombinant haplotype, the coefficient of selection required drastically increases to 0.573. However, in all scenarios of this model, selection can only maintain polymorphism and LD if the non‐recombinant allele frequencies are identical (*g*_*AB*_ = *g*_*ab*_; see Figure 4A,B). If this condition is not met, the less frequent haplotype is eventually lost (Figure 4C,D). This occurs because the less frequent non‐recombinant haplotype is disproportionately affected by the loss of alleles to recombinant haplotypes through recombination, as it is more concentrated in double heterozygous compared to the other non‐recombinant haplotype. The addition of overdominance in the second model increases the system's stability. Figure 5 shows some properties of this scenario. Since *s*_*r*_ represents the reduction in fitness of genotypes with a single recombinant chromosome, we set *s*_*r*_ to 0.02035 (i.e., 50% of the estimated coefficient of selection against genotypes carrying two recombinant chromosomes). Starting the simulation with initial frequencies of the non‐recombinant haplotypes at opposite extremes (e.g., *g*_*AB*_ = 0.01 and *g*_*ab*_ = 0.99), *r* = 0.0015 and *s*_*ho*_ = *s*_*r*_ = 0.02035 led *g*_*AB*_ and *g*_*ab*_ to intermediate frequencies with a small excess of heterozygotes (Figure 5A). An excess of double heterozygous comparable to that observed for DA‐PA0 (mean *D*_AB_ ≈ 0.025) happened when *s*_*ho*_ reached ~0.05 (Figure 5B). The system became unstable under very weak overdominance (*s*_*ho*_ < 0.0008—Figure 5C) and high recombination rates (e.g., *r* = 0.10 and *s*_*ho*_ = 0.015—arbitrary values—Figure 5D).

## 4 Discussion

Since the first large‐scale investigation into the population dynamics of chromosome II inversions of *D. mediopunctata* at the Parque Nacional do Itatiaia in the 1980s, several studies have gathered evidence demonstrating that this system is a target of natural selection (e.g., Klaczko, Otto, and Peixoto 1990; Peixoto and Klaczko 1991; Ananina et al. 2004; Batista et al. 2018). Many aspects of this system have parallels in other studies. For example, various *Drosophila* species exhibit geographic and seasonal patterns of inversion frequency variation, with some polymorphisms maintained by overdominance and epistasis, aligning with Dobzhansky's coadaptation hypothesis (reviewed in Singh 2018). Complex polymorphisms involving linked, non‐overlapping inversions have been observed in species like *D. robusta*, *D. subobscura*, and *D. pseudoobscura* (Levitan and Etges 2005; Pegueroles et al. 2016; Fuller et al. 2020). Furthermore, the long‐term maintenance of LD aspect is not exclusive to the *Drosophila* genus or chromosome inversions: for instance, Webster (1973) reported long‐term maintenance of LD in the salamander *Plethodon cinereus*; and Barrière and Félix (2007) found evidence of selection maintaining LD among microsatellite loci in *C. elegans*. The inversions of *D. mediopunctata* contribute to the empirical investigation of complex polymorphisms by demonstrating a specific combination of multiple superimposed layers: inversion polymorphism and thermal adaptation, long‐term LD maintenance, and overdominance.

Our first aim was to determine whether the LD observed 30 years ago had changed after more than 300 generations by comparing current LD values with the expected neutral decline toward equilibrium. Our results show that such a decline did not occur. Not only are the present LD values remained virtually unchanged for the past 30 years, but their distribution pattern is also akin (Figure 1). Genetic drift may occasionally generate LD (Ohta and Kimura 1969). Batista et al. (2018) showed that microsatellite markers show low population genetic structure (FST = 0.022) and high migration (with Nm varying between 7.9 and 11.7) in a set of samples, including the Parque Nacional do Itatiaia and forest fragments with > 200 km between them. This, coupled with the consistency of the LD patterns over three decades, allows us to dismiss genetic drift as a reasonable cause for the LD observed. Two major scenarios could explain the observed LD patterns: (a) Adaptive differentiation with migration/admixture (see Kirkpatrick and Barton 2006; Charlesworth and Barton 2018) or (b) Positive epistasis among genes associated with distal and proximal inversions. The LD pattern analysis suggests that the first scenario is unlikely. According to Thomson and Klitz (1987), the pattern of LD distribution caused by selection is only mimicked by migration/admixture under strict proportionality of allele frequencies in the populations involved. While this may apply to one‐time studies that could capture a fortuitous moment of population admixture with such proportionality, it is doubtful that such a system would keep the apparent long‐term stability that our results suggest Consequently, our results align with the hypothesis of recurrent selection against recombinant chromosomes.

Next, we moved to a broader level of organization by examining how entire haplotypes associate to form inversion karyotypes rather than focusing solely on the association of alleles into haplotypes. The analysis of all 2010s collections, combining samples taken at different moments in the seasonal cycle, revealed a trend toward a deficiency of homokaryotypes for PA and PB‐sharing haplotypes and an excess of the heterokaryotype formed by the combination of these two inversion haplotypes. However, the most revealing results emerged when we reanalyzed our dataset alongside Klaczko, Otto, and Peixoto (1990) organizing the data by seasonal cycle prior to analysis. This approach revealed seemingly highly stable dynamics operating at the karyotype level: in both datasets, collected over 300 generations apart, the excess of the most common heterokaryotypes alternates between seasons. Specifically, *D*_PB/PA_ is farther from zero at the end of the cold season, and at the end of the warm season, it approaches zero. *D*_PB/PC_, in turn, shows the opposite pattern, getting farther from zero at the end of the warm season and closer to zero at the end of the cold season (Figure 2). The homokaryotypic counterparts show the same qualitative pattern (Figure S2). These results suggest that the seasonal variation described for haplotypes DA‐PA0 and DS + DP/PC0 by Ananina et al. (2004) may represent a case of what we might call “seasonal heterosis.” In this conjecture, the heterokaryotype DI‐PB0/DA‐PA0 is favored during colder months, increasingly deviating from HWE toward the end of the season, while the heterokaryotype DI‐PB0/DS (or DV or DP)‐PC0 is favored during warmer months, similarly resulting in excess by the end of the warm season. While there is substantial evidence that the DA‐PA0 haplotype is favored during cold months and the PC0 haplotypes during warm months (Ananina et al. 2004), it appears that selection consistently favors the heterokaryotypes formed by these haplotypes in combination with DI‐PB0, rather than their corresponding homokaryotypes.

We then explored the parametric space to evaluate which values could account for the striking stability of this dynamic system. Although the exploratory manipulation of the deterministic models used here does not allow for broad generalization, our findings align with the conclusions of analytical approaches to the two‐locus, two‐allele system. Internal equilibrium—i.e., one that maintains polymorphism in each locus—strongly depends on a balance between LD, recombination rate, and epistatic selection (e.g., Lewontin and Kojima 1960; Hastings 1981). Moreover, the stability of internal equilibrium was conditional on the presence of overdominance (see Pontz, Hofbauer, and Bürger 2018). Our epistatic fitness scheme differs from previous models by incorporating a position effect, where $w_{\mathrm{AB}/\mathrm{ab}}\neq w_{\mathrm{Ab}/\mathrm{aB}}$. While this addition is unlikely to significantly affect our conclusions, given the already low frequency of double recombinant heterozygotes in the population, it is a biologically plausible consideration. Although the molecular nature of epistatic interactions remains elusive, strong evidence links regulatory interactions to LD blocks (Said et al. 2018), even if this does not imply a direct relationship between chromatin interactions and LD (Whalen and Pollard 2019).

In double heterozygotes, recombination is nearly wholly suppressed, both within and between inversions (Peixoto and Klaczko 1991; Ananina et al. 2004; Hatadani and Klaczko 2018), suggesting that the production of recombinant chromosomes in natural populations is very low. This suppression allows a relatively small selection coefficient to maintain the observed LD once the population reaches the equilibrium frequency of recombinant chromosomes (${\hat{g}}_{R}$), assuming weak genetic drift in this population (Batista et al. 2018). With no dominance, a selection coefficient of ~0.0407 would be sufficient to counterbalance the effect of recombination for the DA‐PA0 haplotype with the observed ${\hat{g}}_{R}≅0.0356$. In a similar system involving non‐overlapping, linked inversions, Fuller et al. (2020, p. 215) also postulated the role of epistatic selection in the long‐term maintenance of LD. However, unlike our findings, they suggest that the highly differentiated SR chromosome might be maintained by a combination of “extensive recombination suppression and strong epistatic selection,” with haploid selection coefficients of up to 0.65.

However, the simple epistatic model alone is unlikely to explain the long‐term persistence of LD in this population, as it depends on the unrealistic assumption of constant equality $g_{\mathrm{AB}}=g_{\mathrm{ab}}$. Not only does genetic drift make this constancy very unlikely, but these inversions also show marked spatial and seasonal variation in Itatiaia (Ananina et al. 2004). Therefore, we explored a slightly more complex scenario in which overdominance at each locus affects the fitness of each inversion karyotype in combination with the presence of a recombinant chromosome. This approach resulted in a much more stable system that maintains polymorphism and LD across a range of parameter setups. However, achieving the observed heterozygous excess for the most common haplotypes $\left(D_{\mathrm{DA}-\mathrm{PA}0/\mathrm{DI}-\mathrm{PB}0}\approx 0.025\right)$ requires moderate selection coefficients $\left(s_{\mathrm{ho}}\sim 0.05\right)$. Given the physical distance between distal and proximal inversions and the size of the arrangements themselves (Ananina et al. 2002), recombination inhibition likely encompasses much, if not all, of the chromosome. Considering the probable homology with the 3R chromosomal arm of *Drosophila melanogaster* (Brianti et al. 2009), it is plausible that the second chromosome of *D. mediopunctata* harbors around 4000 potential genic targets of selection (Hoskins et al. 2015). A substantial part of these sequences is likely to be indirectly affected by natural selection, as LD does not tend to be uniformly distributed within inverted chromosomes (Schaeffer et al. 2003; Wallace, Detweiler, and Schaeffer 2013; Rane et al. 2015; Pegueroles et al. 2016). Hence, selection on the second chromosome inversion haplotypes of *D. mediopunctata* potentially impacts large genic blocks, affecting multiple traits.

Dobzhansky's coadaptation hypothesis (Dobzhansky and Levene 1948; Dobzhansky 1970) makes two key predictions about the interactions among loci within and between inverted sequences. First, inversions should consist of coadapted alleles that interact positively among themselves. Second, the interaction among coadapted alleles (or polygenic complexes) from chromosomes with different chromosomal arrangements should increase fitness, provided such arrangements are from the same local population. While our results conform to Dobzhansky's coadaptation hypothesis, we still lack data regarding genetic differentiation among arrangements from different populations of *D. mediopunctata*, as well as experimental evaluation of the adaptive values of these “hybrid” heterozygotes. Moreover, results obtained 30 years apart with no intermediate observations advise for caution and future surveys.

The maintenance of preferential haplotype associations and of the signal of karyotype deviations over decades denotes a remarkable case of natural selection acting on a complex inversion polymorphism in a natural population, seemingly fitting all requisites outlined by Lewontin and Kojima (1960). Our study strengthens the hypothesis that the long‐term maintenance of LD and inversion polymorphism is driven by natural selection and demonstrates that this system offers a unique opportunity to investigate the various factors and forces shaping inversion evolution (Faria et al. 2019). The second chromosome of *D. mediopunctata* corresponds to Muller element E (Brianti, Ananina, and Klaczko 2013), which is known to harbor candidate genes for thermal adaptation, such as *Hsp70* (e.g., Pegueroles et al. 2013), and has been shown to affect traits like body size (Hatadani and Klaczko 2008) and abdominal pigmentation (Hatadani et al. 2004). Further studies tackling the fitness components of heterokaryotypes and the genetic content of these chromosomal inversions should help clarify the evolutionary dynamics of this inversion system.

## Author Contributions

**Fabiana Uno:** conceptualization (equal), data curation (lead), formal analysis (equal), investigation (lead), methodology (equal), project administration (lead), software (lead), supervision (equal), validation (equal), visualization (equal), writing – original draft (lead), writing – review and editing (equal). **Felipe Bastos Rocha:** conceptualization (supporting), formal analysis (equal), investigation (supporting), methodology (equal), validation (equal), visualization (equal), writing – review and editing (equal). **Louis Bernard Klaczko:** conceptualization (equal), funding acquisition (lead), methodology (supporting), project administration (supporting), resources (lead), supervision (lead), writing – review and editing (supporting).

## Conflicts of Interest

The authors declare no conflicts of interest.

## Supporting information


File S1

File S2

## Data Availability

File S1 and S2 contains the python codes used to run the simulations. The authors affirm that all data necessary for confirming the conclusions of the article are present within the article, appendix, figures, and tables.

## References

[ece370443-bib-0001] Ananina, G. , A. A. Peixoto , B. C. Bitner‐Mathé , et al. 2004. “Chromosomal Inversion Polymorphism in *Drosophila mediopunctata*: Seasonal, Altitudinal, and Latitudinal Variation.” Genetics and Molecular Biology 27: 61–69.

[ece370443-bib-0002] Ananina, G. , A. A. Peixoto , W. N. Souza , and L. B. Klaczko . 2002. “Polytene Chromosome Map and Inversion Polymorphism in *Drosophila mediopunctata* .” Memórias do Instituto Oswaldo Cruz 97: 691–694. 12219137 10.1590/s0074-02762002000500019

[ece370443-bib-0003] Anderson, A. R. , A. A. Hoffmann , S. W. Mckechnie , P. A. Umina , and A. R. Weeks . 2005. “The Latitudinal Cline in the In(3R)Payne Inversion Polymorphism Has Shifted in the Last 20 Years in Australian *Drosophila melanogaster* Populations.” Molecular Ecology 14: 851–858. 15723676 10.1111/j.1365-294X.2005.02445.x

[ece370443-bib-0004] Arnold, J. 1981. “Statistics of Natural Populations. I: Estimating an Allele Probability in Cryptic Fathers With a Fixed Number of Offspring.” Biometrics 37: 495–504.

[ece370443-bib-0005] Asmussen, M. A. , and M. T. Clegg . 1982. “Rates of Decay of Linkage Disequilibrium Under Two‐Locus Models of Selection.” Journal of Mathematical Biology 14, no. 1: 37–70.

[ece370443-bib-0006] Barrière, A. , and M. A. Félix . 2007. “Temporal Dynamics and Linkage Disequilibrium in Natural *Caenorhabditis elegans* Populations.” Genetics 176, no. 2: 999–1011. 17409084 10.1534/genetics.106.067223 PMC1894625

[ece370443-bib-0007] Batista, M. R. , R. E. Penha , S. H. Sofia , and L. B. Klaczko . 2018. “Comparative Analysis of Adaptive and Neutral Markers of *Drosophila mediopunctata* Populations Dispersed Among Forest Fragments.” Ecology and Evolution 8, no. 24: 12681–12693. 30619573 10.1002/ece3.4696 PMC6308856

[ece370443-bib-0008] Brianti, M. T. , G. Ananina , and L. B. Klaczko . 2013. “Differential Occurrence of Chromosome Inversion Polymorphisms Among Muller's Elements in Three Species of the Tripunctata Group of *Drosophila*, Including a Species With Fast Chromosomal Evolution.” Genome 56, no. 1: 17–26. 23379335 10.1139/gen-2012-0074

[ece370443-bib-0009] Brianti, M. T. , G. Ananina , S. M. Recco‐Pimentel , and L. B. Klaczko . 2009. “Comparative Analysis of the Chromosomal Positions of rDNA Genes in Species of the Tripunctata Radiation of *Drosophila* .” Cytogenetic and Genome Research 125, no. 2: 149–157. 19729919 10.1159/000227840

[ece370443-bib-0011] Carson, H. L. 1958. “The Population Genetics of *Drosophila robusta* .” Advances in Genetics 9: 1–40. 13520438 10.1016/s0065-2660(08)60158-3

[ece370443-bib-0012] Carvalho, A. B. , A. A. Peixoto , and L. B. Klaczko . 1989. “Sex‐Ratio in *Drosophila mediopunctata* .” Heredity 62: 425–428.

[ece370443-bib-0013] Cavasini, R. , K. A. Carvalho , and L. B. Klaczko . 2010. “Recombination in *Drosophila mediopunctata* .” Drosophila Information Service 93: 122–124.

[ece370443-bib-0014] Charlesworth, B. , and N. H. Barton . 2018. “The Spread of an Inversion With Migration and Selection.” Genetics 208, no. 1: 377–382. 29158424 10.1534/genetics.117.300426 PMC5753870

[ece370443-bib-0015] Chinnici, J. P. 1971. “Modification of Recombination Frequency in *Drosophila*. II. The Polygenic Control of Crossing Over.” Genetics 69, no. 1: 85–96. 5002415 10.1093/genetics/69.1.85 PMC1212691

[ece370443-bib-0016] Crown, K. N. , D. E. Miller , J. Sekelsky , and R. S. Hawley . 2018. “Local Inversion Heterozygosity Alters Recombination Throughout the Genome.” Current Biology 28, no. 18: 2984–2990. 30174188 10.1016/j.cub.2018.07.004 PMC6156927

[ece370443-bib-0017] Dobzhansky, T. 1947. “Adaptive Changes Induced by Natural Selection in Wild Populations of *Drosophila* .” Evolution 1: 1–16.

[ece370443-bib-0019] Dobzhansky, T. 1970. Genetics of the Evolutionary Process. New York: Columbia University Press.

[ece370443-bib-0020] Dobzhansky, T. , and H. Levene . 1948. “Operation of Natural Selection in Wild Populations. XVII. Proof of Operation of Natural Selection in Wild Populations of *Drosophila pseudoobscura* .” Genetics 33: 537–547. 18100287 10.1093/genetics/33.6.537 PMC1209427

[ece370443-bib-0021] Etges, W. J. , K. L. Arbuckle , and M. A. X. Levitan . 2006. “Long‐Term Frequency Shifts in the Chromosomal Polymorphisms of *Drosophila robusta* in the Great Smoky Mountains.” Biological Journal of the Linnean Society 88, no. 1: 131–141.

[ece370443-bib-0023] Faria, R. , K. Johannesson , R. K. Butlin , and A. M. Westram . 2019. “Evolving inversions.” Trends in Ecology & Evolution 34: 239–248. 30691998 10.1016/j.tree.2018.12.005

[ece370443-bib-0024] Fontdevila, A. , C. Zapata , G. Alvarez , L. Sanchez , J. Méndez , and I. Enriquez . 1983. “Genetic Coadaptation in the Chromosomal Polymorphism of *Drosophila subobscura*. I. Seasonal Changes of Gametic Disequilibrium in a Natural Population.” Genetics 105: 935–955. 17246183 10.1093/genetics/105.4.935 PMC1202235

[ece370443-bib-0025] Fuller, Z. L. , G. D. Haynes , S. Richards , and S. W. Schaeffer . 2016. “Genomics of Natural Populations: How Differentially Expressed Genes Shape the Evolution of Chromosomal Inversions in *Drosophila pseudoobscura* .” Genetics 204, no. 1: 287–301. 27401754 10.1534/genetics.116.191429 PMC5012393

[ece370443-bib-0026] Fuller, Z. L. , S. A. Koury , C. J. Leonard , et al. 2020. “Extensive Recombination Suppression and Epistatic Selection Causes Chromosome‐Wide Differentiation of a Selfish Sex Chromosome in *Drosophila pseudoobscura* .” Genetics 216, no. 1: 205–226. 32732371 10.1534/genetics.120.303460 PMC7463281

[ece370443-bib-0027] Gillespie, J. H. 2010. Population Genetics: A Concise Guide. Baltimore: Johns Hopkins University Press.

[ece370443-bib-0028] Gong, W. J. , K. S. McKim , and R. S. Hawley . 2005. “All Paired Up With No Place to Go: Pairing, Synapsis, and DSB Formation in a Balancer Heterozygote.” PLoS Genetics 1: e67. 16299588 10.1371/journal.pgen.0010067 PMC1285065

[ece370443-bib-0029] Hastings, A. 1981. “Disequilibrium, Selection, and Recombination: Limits in Two‐Locus, Two‐Allele Models.” Genetics 98, no. 3: 659–668. 7333456 10.1093/genetics/98.3.659 PMC1214465

[ece370443-bib-0030] Hatadani, L. M. , J. C. R. Baptista , W. N. Souza , and L. B. Klaczko . 2004. “Colour Polymorphism in *Drosophila mediopunctata*: Genetic (Chromosomal) Analysis and Nonrandom Association With Chromosome Inversions.” Heredity 93, no. 6: 525–534. 15305174 10.1038/sj.hdy.6800544

[ece370443-bib-0031] Hatadani, L. M. , and L. B. Klaczko . 2008. “Shape and Size Variation on the Wing of *Drosophila mediopunctata*: Influence of Chromosome Inversions and Genotype‐Environment Interaction.” Genetica 133, no. 3: 335–342. 17952608 10.1007/s10709-007-9217-7

[ece370443-bib-0032] Hatadani, L. M. , and L. B. Klaczko . 2018. “Recombination Rate Between Inversions in the Distal and Proximal Regions of the Second Chromosome of *Drosophila mediopunctata* .” Drosophila Information Service 101: 55–60.

[ece370443-bib-0033] Hoskins, R. A. , J. W. Carlson , K. H. Wan , et al. 2015. “The Release 6 Reference Sequence of the *Drosophila melanogaster* Genome.” Genome Research 25, no. 3: 445–458. 25589440 10.1101/gr.185579.114 PMC4352887

[ece370443-bib-0034] Joron, M. , L. Frezal , R. T. Jones , et al. 2011. “Chromosomal Rearrangements Maintain a Polymorphic Supergene Controlling Butterfly Mimicry.” Nature 477: 203–206. 21841803 10.1038/nature10341 PMC3717454

[ece370443-bib-0036] Kennington, W. J. , L. Partridge , and A. A. Hoffmann . 2006. “Patterns of Diversity and Linkage Disequilibrium Within the Cosmopolitan Inversion In(3R)Payne in *Drosophila melanogaster* Are Indicative of Coadaptation.” Genetics 172: 1655–1663. 16322502 10.1534/genetics.105.053173 PMC1456293

[ece370443-bib-0037] Kirkpatrick, M. , and N. Barton . 2006. “Chromosome Inversions, Local Adaptation and Speciation.” Genetics 173: 419–434. 16204214 10.1534/genetics.105.047985 PMC1461441

[ece370443-bib-0038] Klaczko, L. B. 1995. “Population Genetics of *Drosophila mediopunctata* .” In Genetics of Natural Populations: The Continuing Importance of Theodosius Dobzhansky, edited by L. Levine , 140–153. New York: Columbia University Press.

[ece370443-bib-0039] Klaczko, L. B. 2006. “Evolutionary Genetics of *Drosophila mediopunctata* .” Genetica 126: 43–55. 16502084 10.1007/s10709-005-1431-6

[ece370443-bib-0040] Klaczko, L. B. , P. A. Otto , and A. A. Peixoto . 1990. “Allele Frequency Estimates When Only Heterozygotes Can Be Recognized: Method of Estimation and Application to the Case of Chromosomal Inversion Polymorphisms in *Drosophila* .” Heredity 64: 263–270. 2341292 10.1038/hdy.1990.32

[ece370443-bib-0041] Koury, S. A. 2023. “Predicting Recombination Suppression Outside Chromosomal Inversions in *Drosophila melanogaster* Using Crossover Interference Theory.” Heredity 130, no. 4: 196–208. 36721031 10.1038/s41437-023-00593-x PMC10076299

[ece370443-bib-0042] Krimbas, C. B. , and E. Zouros . 1969. “Crossing‐Over Suppression Between Linked but Non‐Overlapping Inversions in *Drosophila subobscura* .” Drosophila Information Service 44: 71–72.

[ece370443-bib-0043] Levitan, M. 1955. “Studies of Linkage in Populations. I. Associations of Second Chromosome Inversions in *Drosophila robusta* .” Evolution 9: 62–74.

[ece370443-bib-0044] Levitan, M. 1961. “Proof of an Adaptive Linkage Association.” Science 134: 1617–1619. 10.1126/science.134.3490.1617-a 14464697

[ece370443-bib-0045] Levitan, M. 1973. “Studies of Linkage in Populations. VI. Periodic Selection for X‐Chromosome Gene Arrangement Combinations.” Evolution 27: 215–225. 28564777 10.1111/j.1558-5646.1973.tb00667.x

[ece370443-bib-0046] Levitan, M. , and W. J. Etges . 2005. “Climate Change and Recent Genetic Flux in Populations of *Drosophila robusta* .” BMC Evolutionary Biology 5: 4. 15636637 10.1186/1471-2148-5-4 PMC548147

[ece370443-bib-0047] Levitan, M. , and F. M. Salzano . 1959. “Studies of Linkage in Populations. III. An Association of Linked Inversions in *Drosophila guaramunu* .” Heredity 13: 243–248.

[ece370443-bib-0048] Lewontin, R. C. 1964. “The Interaction of Selection and Linkage. I. General Considerations; Heterotic Models.” Genetics 49, no. 1: 49–67. 17248194 10.1093/genetics/49.1.49 PMC1210557

[ece370443-bib-0049] Lewontin, R. C. , and K. I. Kojima . 1960. “The Evolutionary Dynamics of Complex Polymorphisms.” Evolution 14, no. 4: 458–472.

[ece370443-bib-0050] Navarro, A. , E. Betrán , A. Barbadilla , and A. Ruiz . 1997. “Recombination and Gene Flux Caused by Gene Conversion and Crossing Over in Inversion Heterokaryotypes.” Genetics 146: 695–709. 9178017 10.1093/genetics/146.2.695 PMC1208008

[ece370443-bib-0051] Navarro‐Dominguez, B. , C. H. Chang , C. L. Brand , C. A. Muirhead , D. C. Presgraves , and A. M. Larracuente . 2022. “Epistatic Selection on a Selfish Segregation Distorter Supergene–Drive, Recombination, and Genetic Load.” eLife 11: e78981. 35486424 10.7554/eLife.78981 PMC9122502

[ece370443-bib-0052] Nomura, T. , S. Suzuki , T. Miyauchi , et al. 2018. “Chromosomal Inversions as a Hidden Disease‐Modifying Factor for Somatic Recombination Phenotypes.” JCI Insight 3, no. 6: e98921. 29563344 10.1172/jci.insight.97595 PMC5926924

[ece370443-bib-0053] Ohta, T. , and M. Kimura . 1969. “Linkage Disequilibrium Due to Random Genetic Drift.” Genetic Research 13: 47–55. 10.1093/genetics/63.1.229 PMC1212334 5365295

[ece370443-bib-0054] Ortiz‐Barrientos, D. , J. Engelstädter , and L. H. Rieseberg . 2016. “Recombination Rate Evolution and the Origin of Species.” Trends in Ecology & Evolution 31, no. 3: 226–236. 26831635 10.1016/j.tree.2015.12.016

[ece370443-bib-0055] Pál, C. , and L. D. Hurst . 2003. “Evidence for Co‐Evolution of Gene Order and Recombination Rate.” Nature Genetics 33, no. 3: 392–395. 12577060 10.1038/ng1111

[ece370443-bib-0056] Pegueroles, C. , C. F. Aquadro , F. Mestres , and M. Pascual . 2013. “Gene Flow and Gene Flux Shape Evolutionary Patterns of Variation in *Drosophila subobscura* .” Heredity 110: 520–529. 23321709 10.1038/hdy.2012.118 PMC3656635

[ece370443-bib-0057] Pegueroles, C. , A. Ferres‐Coy , M. Marti‐Solano , C. F. Aquadro , M. Pascual , and F. Mestres . 2016. “Inversions and Adaptation to the Plant Toxin Ouabain Shape DNA Sequence Variation Within and Between Chromosomal Inversions of *Drosophila subobscura* .” Scientific Reports 6: 23772. 27029337 10.1038/srep23754 PMC4815013

[ece370443-bib-0058] Pegueroles, C. , V. Ordóñez , F. Mestres , and M. Pascual . 2010. “Recombination and Selection in the Maintenance of the Adaptive Value of Inversions.” Journal of Evolutionary Biology 23: 2709–2717. 20964762 10.1111/j.1420-9101.2010.02136.x

[ece370443-bib-0059] Peixoto, A. A. , and L. B. Klaczko . 1991. “Linkage Disequilibrium Analysis of Chromosomal Inversion Polymorphisms of *Drosophila* .” Genetics 129: 773–777. 1752420 10.1093/genetics/129.3.773 PMC1204744

[ece370443-bib-0060] Pontz, M. , J. Hofbauer , and R. Bürger . 2018. “Evolutionary Dynamics in the Two‐Locus Two‐Allele Model With Weak Selection.” Journal of Mathematical Biology 76: 151–203. 28547213 10.1007/s00285-017-1140-7 PMC5754571

[ece370443-bib-0061] Puig, M. , M. Cáceres , and A. Ruiz . 2004. “Silencing of a Gene Adjacent to the Breakpoint of a Widespread *Drosophila* Inversion by a Transposon‐Induced Antisense RNA.” Proceedings of the National Academy of Sciences of the United States of America 101, no. 24: 9013–9018. 15184654 10.1073/pnas.0403090101 PMC428464

[ece370443-bib-0062] Rane, R. V. , L. Rako , M. Kapun , S. F. Lee , and A. A. Hoffmann . 2015. “Genomic Evidence for Role of Inversion 3RP of *Drosophila melanogaster* in Facilitating Climate Change Adaptation.” Molecular Ecology 24: 2423–2432. 25789416 10.1111/mec.13161

[ece370443-bib-0063] Rybnikov, S. R. , Z. Frenkel , S. Hübner , D. B. Weissman , and A. B. Korol . 2023. “Modeling the Evolution of Recombination Plasticity: A Prospective Review.” BioEssays 45, no. 8: 2200237. 10.1002/bies.202200237 37246937

[ece370443-bib-0064] Said, I. , A. Byrne , V. Serrano , C. Cardeno , C. Vollmers , and R. Corbett‐Detig . 2018. “Linked Genetic Variation and Not Genome Structure Causes Widespread Differential Expression Associated With Chromosomal Inversions.” Proceedings of the National Academy of Sciences of the United States of America 115, no. 21: 5492–5497. 29735663 10.1073/pnas.1721275115 PMC6003460

[ece370443-bib-0065] Schaeffer, S. W. , M. P. Goetting‐Minesky , M. Kovacevic , et al. 2003. “Evolutionary Genomics of Inversions in *Drosophila pseudoobscura*: Evidence for Epistasis.” Proceedings of the National Academy of Sciences of the United States of America 100: 8319–8324. 12824467 10.1073/pnas.1432900100 PMC166227

[ece370443-bib-0066] Singh, B. N. 2008. “Chromosome Inversions and Linkage Disequilibrium in *Drosophila* .” Current Science 95, no. 4: 459–464.

[ece370443-bib-0067] Singh, B. N. 2018. “Dobzhansky's Concept of Genetic Coadaptation: *Drosophila ananassae* Is an Exception to This Concept.” Journal of Genetics 97, no. 4: 1039–1046. 30262716

[ece370443-bib-0068] Stapley, J. , P. G. Feulner , S. E. Johnston , A. W. Santure , and C. M. Smadja . 2017. “Variation in Recombination Frequency and Distribution Across Eukaryotes: Patterns and Processes.” Philosophical Transactions of the Royal Society, B: Biological Sciences 372, no. 1736: 20160455. 10.1098/rstb.2016.0455 PMC5698618 29109219

[ece370443-bib-0069] Sturtevant, A. H. 1926. “A Crossover Reducer in *Drosophila melanogaster* Due to Inversion of a Section of the Third Chromosome.” Genetics 11: 135–154.

[ece370443-bib-0070] Thomson, G. , and W. Klitz . 1987. “Disequilibrium Pattern Analysis. I. Theory.” Genetics 116: 623–632. 3623083 10.1093/genetics/116.4.623 PMC1203175

[ece370443-bib-0071] Wallace, A. G. , D. Detweiler , and S. W. Schaeffer . 2013. “Molecular Population Genetics of Inversion Breakpoint Regions in *Drosophila pseudoobscura* .” G3: Genes, Genomes, Genetics 3: 1151–1163. 23665879 10.1534/g3.113.006122 PMC3704243

[ece370443-bib-0072] Webster, T. P. 1973. “Adaptive Linkage Disequilibrium Between Two Esterase Loci of a Salamander.” Proceedings of the National Academy of Sciences of the United States of America 70, no. 4: 1156–1160. 4515614 10.1073/pnas.70.4.1156 PMC433447

[ece370443-bib-0073] Weir, B. S. 1996. Genetic Data Analysis II: Methods for Discrete Population Genetic Data. Sunderland, MA: Sinauer Associates.

[ece370443-bib-0074] Wellenreuther, M. , and L. Bernatchez . 2018. “Eco‐Evolutionary Genomics of Chromosomal Inversions.” Trends in Ecology & Evolution 33, no. 6: 427–440. 29731154 10.1016/j.tree.2018.04.002

[ece370443-bib-0075] Whalen, S. , and K. S. Pollard . 2019. “Most Chromatin Interactions Are Not in Linkage Disequilibrium.” Genome Research 29, no. 3: 334–343. 30617125 10.1101/gr.238022.118 PMC6396425

[ece370443-bib-0076] White, N. J. , R. R. Snook , and I. Eyres . 2020. “The Past and Future of Experimental Speciation.” Trends in Ecology & Evolution 35, no. 1: 10–21. 31522756 10.1016/j.tree.2019.08.009

[ece370443-bib-0077] Winbush, A. , and N. D. Singh . 2022. “Variation in Fine‐Scale Recombination Rate in Temperature‐Evolved *Drosophila melanogaster* Populations in Response to Selection.” G3: Genes, Genomes, Genetics 12, no. 10: jkac208. 35961026 10.1093/g3journal/jkac208 PMC9526048

