# Supplementary material for: Long‐Term Maintenance of Complex Chromosomal Inversion Polymorphism in Drosophila mediopunctata
Source: Ecol Evol. 2024 Oct 27;14(10):e70443. doi: 10.1002/ece3.70443 (PMC11513201; doi:10.1002/ece3.70443)
Supplement: Supplementary file 2 — File S2 [file ECE3-14-e70443-s002.docx]

*The following supporting information accompanies the article*

**Long-term maintenance of complex chromosomal inversion polymorphism in *Drosophila mediopunctata***

**Fabiana Uno^1*^, Felipe Bastos Rocha**^2^**, Louis Bernard Klaczko^3^**

*corresponding author: fabiana.uno@ufrj.br

**Section 1.**

**OFFSPRING VIABILITY AND FECUNDITY IN HETEROKARYOTIPIC FEMALES**

To investigate whether the low recombination rate observed in double heterokaryotypic individuals, as inferred from adult morphological markers, was due to the high mortality of recombinants at early developmental stages, we analyzed the fertility components and offspring viability of homozygous and heterozygous individuals for the two most common haplotypes (combination of distal and proximal inversions) in *D. mediopunctata*. We categorized the fitness composite traits analyzed in this work into two groups: i) juvenile fitness, which includes embryonic viability, egg-to-pupa survival, and pupa-to-adult survival, and ii) adult fitness, represented here by female fecundity.

**Fly stocks and crossings**

The two standard laboratory strains of *D. mediopunctata* used in this study are homozygous for different combinations of non-overlapping distal and proximal inversions. The first strain, ITC229ET (designated Ho^DIDI^), is homozygous for the DI-PB0 haplotype, while the second strain, ITA24P (designated Ho^DADA^), is homozygous for the DA-PA0 haplotype. To generate the F_1_ strain (named F_1_^DADI^), we made a cross between ITC229ET females and ITA24P males. The resulting heterokaryotypic F_1_ females (F_1_^DADI^) were then backcrossed to ITC2229ET males to produce the offspring BC^DADI X DIDI^ and crossed to F_1_ males to produce the offspring F_2_^DADI X DADI^. Finally, ITC229ET females were crossed to F_1_ males to produce the offspring BC^DIDI X DADI^. Details on the crossings performed are shown in Table A1 and Figure A1. Strains identified as “recombinant” represent the crossings that could potentially yield recombinant individuals among their offspring, while “non-recombinant” strains refer to the crosses where no recombinant individual are expected to be produced. All flies used in this study were aged 4-6 days at 18^o^C under a 12:12 light: dark cycle.

**Table A1.** Crosses to produce the homokaryotypic parental lines and the heterokaryotypic F_1_

| **Female parent** | **Male parent** | **Offspring** | **Expected** |
| --- | --- | --- | --- |
| Ho^DIDI^ | Ho^DIDI^ | Ho^DIDI X DIDI^ | Non-recombinant |
| Ho^DADA^ | Ho^DADA^ | Ho^DADA X DADA^ | Non-recombinant |
| Ho^DADA^ | Ho^DIDI^ | F_1_ ^DADI^ | Non-recombinant |
| F_1_ ^DADI^ | Ho^DIDI^ | BC^DADI X DIDI^ | Recombinant |
| Ho^DIDI^ | F_1_ ^DADI^ | BC^DIDI X DADI^ | Non-recombinant |
| F_1_ ^DADI^ | F_1_ ^DADI^ | F_2_ ^DADI X DADI^ | Recombinant |

Ho^DIDI^ and Ho^DADA^ stand for the homokaryotypic lines ITC229ET and ITA24P, respectively. F_1_^DADI^ stands for the heterokaryotypic F_1_ generated by the cross between the two homokaryotypic lines, whereas BC^DADI X DIDI^ and BC^DIDI X DADI^ stand for the offspring produced by the reciprocal backcross between the heterokaryotypic F_1_ and the parental strain ITC229ET. The strain F_2_ ^DADI X DADI^ represents the offspring yielded by the cross among F_1_^DADI^ individuals.


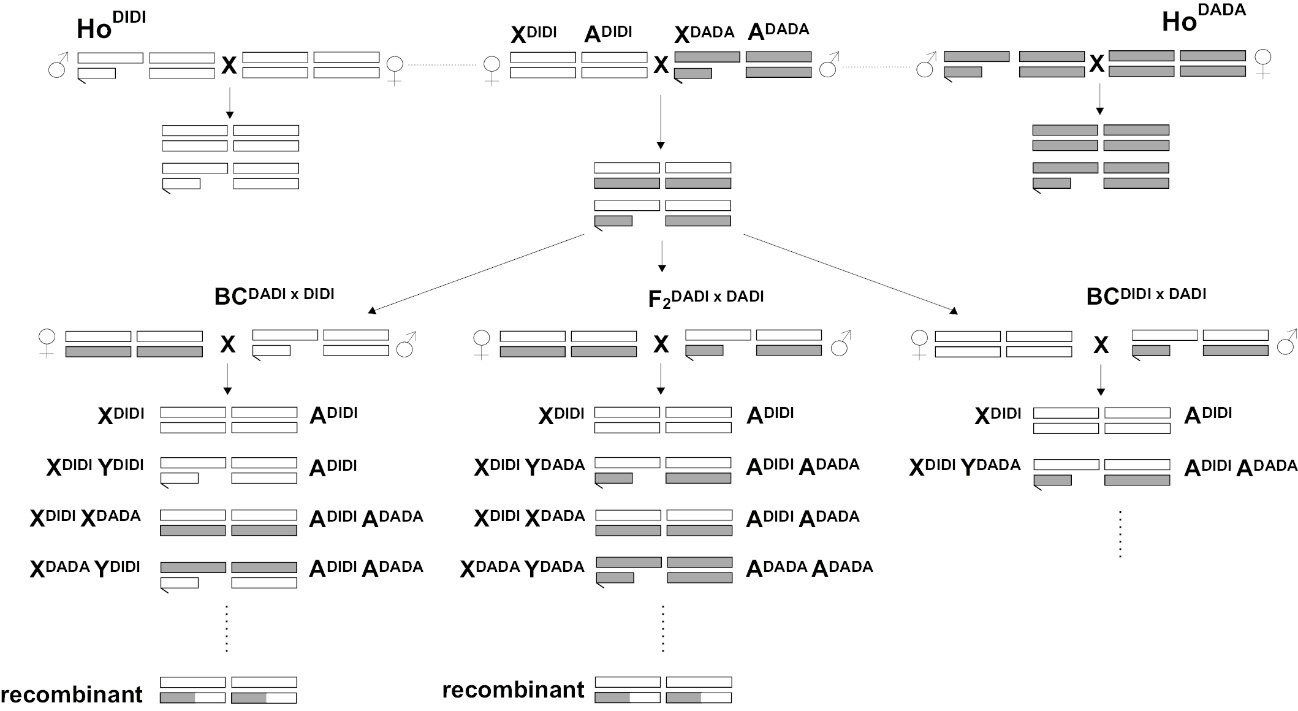


**Figure A1.** Crosses to produce the homokaryotypic parental lines and the heterokaryotypic F_1_ and the experimental offsprings BC^DIDI X DADI^, BC^DADI X DIDI^, and F_2_ ^DADI X DADI^.

**Pre-adult viability**

For the egg-to-pupa and egg-to-adult viability tests, we mass-crossed 100 mature males and 200 virgin females (aged 4 to 6 days) in vials containing trimeveledon culture medium (Carvalho et al 1989). After 24-hours, the flies were transferred to empty half-pint bottles containing slides with agar and yeast paste, which we replaced daily. We transferred batches of 10 eggs to 70 ml vials containing 5ml of culture medium until we completed 160 vials per crossing. The eggs were left to hatch and develop at 18^o^C. We inferred relative viability as the proportion of eggs that successfully developed into pupae and emerged as adult individuals per cross (Iriarte and Hasson., 2000). Preliminary results on the effects of population density on the viability of the standard homokaryotypic lines (Ho^DIDI^ and Ho^DADA^) showed no significant change of viability at densities varying from 2 to 40 eggs per 5ml of medium (Fig A2). Thus, we expect no substantial effect of competition in our egg-to-adult viability tests.


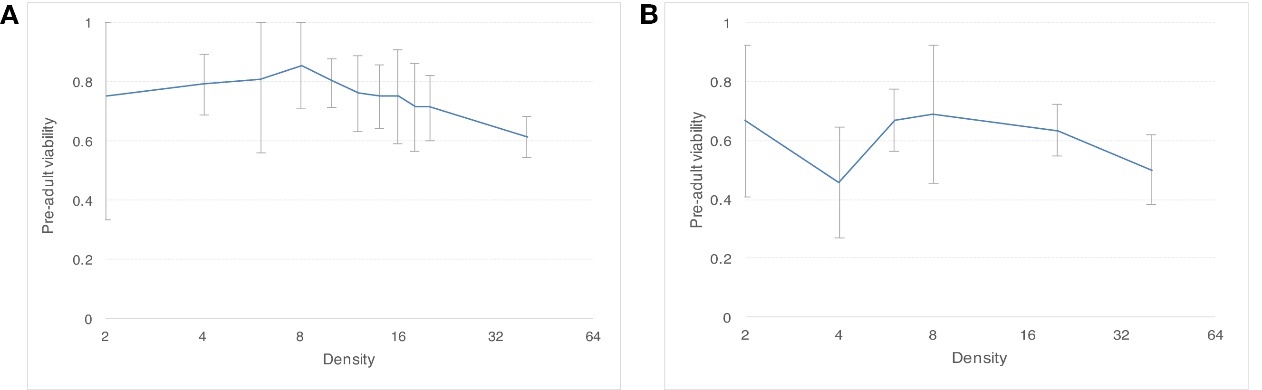


**Figure A2. Pre-adult viability of the homokaryotypic strains Ho^DIDI^ and Ho^DADA^ at various densities.** Each value shown is the means of six replicates. Error bars indicate standard deviation among replicates. (A) Pre-adult viability for the homokaryotypic strain Ho^DIDI^. Despite the seeming reduction in average viability at the density of 40 eggs per vial, a test of homogeneity between densities showed no significant effect on viability over the range of densities tested (X^2^ = 3.79; *d.f*.: 10*;* p > 0.05; for test details, see (27)). (B) We found similar results for the homokaryotypic strain Ho^DADA^ (X^2^= 1.97; *d.f.:* 5; p > 0.05). We conducted all experiments at a constant temperature of 18^o^C.

For the egg-to-larva viability test, egg collections were preceded by 3 to 4-hour egg-laying intervals. We transferred sets of 1000 eggs to Petri dishes filled with 1% agar medium and stored them at 18^o^C. First instar larvae were scored every 24 hours for up to 72 hours. During the analysis, we did not differentiate between inviable and unfertilized eggs; all non-hatching eggs were classified as inviable.

We conducted all the viability experiments simultaneously, except for the evaluation of the offsprings Ho^DADA^ and F_2_^DADI X DADI^ offspring, whose tests we conducted two generations apart from the other groups. Table A2 shows two values of egg-to-larva percentage for the parental strain Ho^DADA^, corresponding to egg samples taken two generations apart. A Chi-square homogeneity test between these two replicates was non-significant (X^2^ = 0.002; *d.f*: 1; p > 0.90), implying that there are no variations in egg viability between measures. Furthermore, we controlled environmental conditions, including temperature, food content and availability, and light regimen, to ensure that the experimental results were directly comparable.

**Fecundity tests**

For the fecundity tests, we assigned 40 females from each cross to individual vials containing 5 ml of a standard medium. The females were allowed to oviposit for 24 hours before being transferred to a fresh vial. We repeated this procedure daily for a total of 5 days. As recommended by McMillan et al. (1970), we aged all flies for eight days before the test, ensuring that the testing period encompassed two days before and two days after the peak egg production of *D mediopunctata* at 18^o^C. The vials were then incubated at 18^o^C to allow development to proceed. We scored the emerging adults daily until no more adults emerged from the vials for at least 72 hours.

An early work by Robertson and Sang (1944) demonstrated that in *D. melanogaster* cultures supplied with excess food and fresh oviposition medium daily, egg viability does not correlate with the total number of eggs laid by a female. Thus, in this study, fecundity is measured by its secondary meaning: the number of adults produced per female per day (Pearl and Parker, 1922).

**Statistical analyses**

To assess the effects of the crossings and developmental stage on overall pre-adult viability, we first tested the variation in viability among the offspring using analyses of variance (ANOVA). We considered the type of *crossing* (HoDIDI, HoDADA, BCDIDI X DADI, BCDADI X DIDI, and F2DADI X DADI) and the *developmental phases*(egg-to-larva, egg-pupa, and pupa-adult) as qualitative explanatory variables; and *viability* (arcsine square-root transformed) of each replicate as the dependent variable. As we found significant effects of both explanatory variables on pre-adult viability, we partitioned the experimental data to better analyze the karyotype's relative viability within developmental stages.

We conducted an analysis of covariance (ANCOVA) for the egg-to-pupa viability tests to assess the effect of three main factors on overall pre-adult viability. These factors were:

1. The female karyotype, which is represented by homo (ITC24P and ITC229ET) and heterokaryotypic (F1) individuals.
2. The average genetic contribution of the standard homokaryotypic strains to the progeny.
3. The presence of recombinant classes in the offspring.

We treated the female karyotype (FK) and the possibility of recombinant classes in the offspring (PRCO) as qualitative explanatory variables. The average genotype of the offspring (AGO) was set as a quantitative explanatory variable and accordingly transformed to a numerical scale of 1 to 0, where 1 is the total genetic contribution of the homokaryotypic ITC229ET strain to the offspring and 0 accounts for no genetic contribution. We adopted a similar procedure for the pupa-to-adult viability and fecundity tests analysis. However, for the latter case, we set the average number of adults produced by each female as the dependent variable. Post-hoc testing was carried out by Tukey's test.

**Results**

Given the experimental design adopted in our analyses, three main factors could influence fecundity and overall pre-adult viability. First is the female karyotype, as we found no significant effect of the male parent on the components of fitness analyzed in this work. Second is the offspring's genotype, which corresponds to the average genetic contribution of each standard homokaryotypic strain to the crossings. Given that the strains used in this study do not share a uniform genetic background, the influence of other chromosomes on viability cannot be overlooked. Third, the female's karyotype determines the potential for recombinant classes in the offspring due to the complete absence of recombination in males (Cavasini et al., 2010). For simplicity, we will refer to this third factor as "recombination" from this point forward.

**Egg-hatchability**

The first question to address is whether the eggs produced by heterokaryotypic females differ in viability from those produced by homokaryotypic females. Table A2 presents the number of hatched eggs counted for each strain within 24, 48, and 72 hours after oviposition. A heterogeneity test showed no significant difference in egg hatchability among the offspring BC^DIDI X DADI^, BC^DADI X DIDI^ and F_2_^DADI X DADI^ and the parental offspring Ho^DIDI^ (X^2^ = 1.97; *d.f.*: 3; p > 0.5); all groups had an egg-to-larva percentage of approximately 90%. However, the proportion of eggs hatched within each 24-hour interval seemed to vary among these four crossings (X^2^ = 865.4; *d.f.*: 3; p < 0.001). The offspring BC^DADI X DIDI^ presented an egg-hatch of 39.8% in the first 24 hours, a value significantly lower than the 57.3% observed for the parental group, the homokaryotic offspring Ho^DIDI^ (X^2^ = 73.62; *d.f.*: 1; p < 0.001). The offspring BC ^DIDI X DADI^ showed an intermediary egg-hatch percentage (47.8%), while the offspring F_2_^DADI X DADI^ showed an almost negligible egg-hatch rate for the same period (0.7%). For the parental line Ho^DADA X DADA^, there seem to be significant differences both in viability and egg-hatching time in contrast with the other strains. It is quite clear from a comparison between both parental lines that Ho^DADA X DADA^ has a significantly lower egg-to-larva viability (74.9%; X^2^ = 72.37; *d.f.*: 1; p < 0.01) and longer embryonic developmental time (2.9% of the viable eggs hatch in the first 24 hours following egg-laying and 94.1% between 24 and 48 hours). Table A3 displays the chi-square values for all groups.

**Table A2.** Egg-hatchability

| **Offspring** | **Hatch from 1000 eggs** | | | | **Egg-to-larva**  **hatchability (%)** |
| --- | --- | --- | --- | --- | --- |
|  | *24h* | *48h* | *72h* | *total* |  |
| Ho^DIDI X DIDI^ | 573 | 321 | 4 | 898 | 89.8 |
| Ho^DADA X DADA^ | 22 | 706 | 22 | 750 | 75.0 |
| Ho^DADA X DADA^ _replicate_ | - | - | - | 749 | 74.9 |
| BC^DADI X DIDI^ | 398 | 512 | 1 | 911 | 91.1 |
| BC^DIDI X DADI^ | 478 | 420 | 1 | 899 | 89.9 |
| F_2_ ^DADI X DADI^ | 7 | 892 | 13 | 912 | 91.2 |

The number of hatched eggs divided by the total number of eggs represents the egg-to-larva hatchability. We conducted two replicates of the parental strain DA-PA0/DA-PA0 (Ho^DADAxDADA^ and Ho^DADAxDADA^_replicate_) to control for experimental error on viability measures taken two generations apart, with no control for egg-hatching time for Ho^DADAxDADA^_replicate_.

**Table A3.** Chi-square for egg-viability and eclosion time

| **Egg - hatchability** | ***d.f.*** | **X^2^** | **p** |
| --- | --- | --- | --- |
| X^2^ between parental lines H_O_^DIDI^ and H_O_^DADA^ | 1 | 75.518 | < 0.001 |
| X^2^ among reciprocal crossings BC^DADI X DIDI^, BC^DIDI X DADI^ and F_2_^DADI X DADI^ | 2 | 1.245 | > 0.1 |
| X^2^ among parental line H_O_^DIDI^ and reciprocal crossings BC^DADI X DIDI^, BC^DIDI X DADI^ and F_2_^DADI X DADI^ | 3 | 1.977 | > 0.1 |
| X^2^ among parental line H_O_^DADA^ and reciprocal crossings BC^DADI X DIDI^, BC^DIDI X DADI^ and F_2_^DADI X DADI^ | 3 | 141.001 | < 0.001 |
|  |  |  |  |
| **Egg - hatchability per 24-hour interval** | ***d.f.*** | **X^2^** | **p** |
| X^2^ between parental lines H_O_^DIDI^ and H_O_^DADA^ | 1 | 656.492 | <0.001 |
| X^2^ among reciprocal crossings BC^DADI X DIDI^, BC^DIDI X DADI^ and F_2_ ^DADI X DADI^ | 2 | 617.320 | <0.001 |
| X^2^ among parental line H_O_^DIDI^ and reciprocal crossings BC^DADI X DIDI^, BC^DIDI X DADI^ and F_2_^DADI X DADI^ | 3 | 865.471 | <0.001 |
| X^2^ among parental line H_O_ ^DADA^ and reciprocal crossings BC^DADI X DIDI^, BC^DIDI X DADI^ and F_2_^DADI X DADI^ | 3 | 1000.637 | <0.001 |

The analysis of covariance (ANCOVA) revealed that neither the female karyotype (F _(1,196)_ = 3.27; p = 0.072) nor the average genotype of the offspring (F _(1,196)_ = 0.930; p = 0.336) had a significant effect on egg-hatchability. Similarly, we found no effect of recombination (F _(1,196)_ = 1.11; p = 0.293; Table A4) on the measured trait. We observed no significant difference in egg-hatchability between the progenies yielded by the homokaryotypic strain Ho^DIDI^ and heterokaryotype females. However, the spawn of females from the homokaryotypic strain Ho^DADA X DADA^ showed significantly lower egg-hatching rates than the other groups (Fig A3).

**Table A4.** Analysis of variance (ANOVA) and Analysis of covariance (ANCOVA) of pre-adult viability and fecundity data.

| **Experiment** | **Components** | **Source** | **d.f.** | **F-ratio** | **P-value** |
| --- | --- | --- | --- | --- | --- |
| Overall pre-adult viability  (ANOVA) | Egg-to-adult | C | 4 | 22.278 | 0.000*** |
|  |  | DS | 1 | 2464.2 | 0.000*** |
|  |  | C x DS | 4 | 23.053 | 0.000*** |
|  |  |  |  |  |  |
| Components of pre-adult viability  (ANCOVA) | Egg-hatchability | FK | 1 | 3.275 | 0.072 |
|  |  | PRCO | 1 | 1.111 | 0.293 |
|  |  | AGO | 1 | 0.930 | 0.336 |
|  |  |  |  |  |  |
|  | Egg-to-pupa | FK | 1 | 8.181 | 0.004** |
|  |  | PRCO | 1 | 0.989 | 0.320- |
|  |  | AGO | 1 | 4.972 | 0.026* |
|  |  |  |  |  |  |
|  | Pupa-to-adult | FK | 1 | 0.096 | 0.756- |
|  |  | PRCO | 1 | 0.026 | 0.871- |
|  |  | AGO | 1 | 5.612 | 0.018* |
|  |  |  |  |  |  |
| Component of Fertility  (ANCOVA) | Female fecundity | FK | 1 | 8.880 | 0.003** |
|  |  | PRCO | 1 | 2.245 | 0.136- |
|  |  | AGO | 1 | 0.199 | 0.656- |

**Abbreviations**: Crossings (C); developmental stage (DS); interaction between crossing and developmental stage (C x DS); female karyotype (FK); average genotype of the offspring (AGO); possibility of recombinant classes in the offspring (PRCO).

*p<0.05; **p<0.01; ***p < 0.001

Heterokaryotype disadvantage due to aneuploidy is not typical, especially for paracentric inversion carriers (White, 1969; Lande, 1984; Coyne et al., 1991). The absence of recombination in males (Cavasini et al., 2010) and the substantial elimination of recombinant products of oogenesis during meiosis (White, 1969) prevent potential deleterious effects of crossing-over encompassing inverted sequences. Accordingly, the absence of significant differences in viability between eggs yielded by either homo or heterokaryotypic females for two sets of linked non-overlapping inversions does not support the hypothesis of embryonic mortality resulting from recombination.


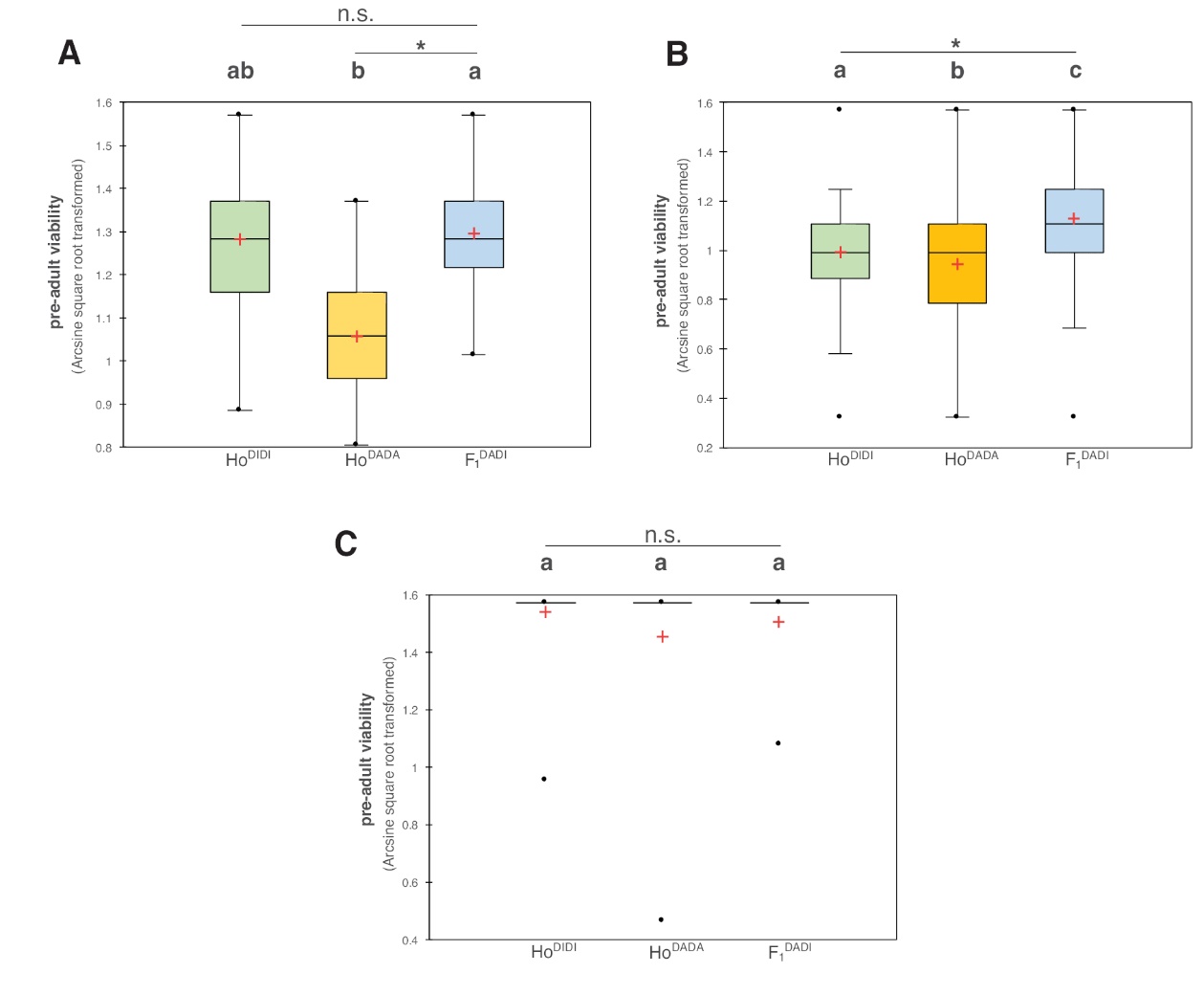
**Figure A3.** Pre-adult viability for different developmental stages. **(**A) Egg hatchability, **(B)**, egg-to-pupa **(C)**, and pupa-to-adult viability. The boxplot contains viability measures for the offspring yielded by homokaryotypic (Ho ^DIDI^, Ho ^DADA^) and heterokaryotypic (F_1_^DADI^) females. The red crosses correspond to the means. Analysis of differences between karyotypes with a confidence interval of 95% (Tukey’s test) is displayed above each group.

**Egg-to-pupa and pupa-to-adult viability**

Collectively, the egg-to-adult viability results unveiled significant effects of developmental phase (F _(1,1584)_ = 2464.29; p < 0.001) and parental karyotype (F _(1,1584)_ = 22.27; p < 0.001) on viability. Moreover, we found significant interactions between the crossings and the developmental phase in which the measures were taken (F _(1,1584)_ = 23.053; p < 0.001; Table A4). Thus, we decided to analyze each of the developmental stages comprised within the juvenile fitness component separately to assess the effects of female karyotype, offspring genotype, and recombination on relative pre-adult viability.

Tables A5 and A6 summarize the results of the egg-to-adult viability tests. Those numbers represent the proportion of eggs that successfully developed into pupae and the percentage of these pupae that later emerged as adults. Vials that showed excessive mold growth and thus could be affecting viability measures were excluded from the analysis. Due to lack of recombination in males of *D. mediopunctata* (Cavasini et al., 2010), crossing-over between distinct karyotypes, and therefore the production of recombinant heterokaryotypes, if present, would only take place in the crossings BC^DADI X DIDI^ and F_2_^DADI X DADI^. Furthermore, as the X chromosome has a minor role in coding for juvenile fitness variation (Chippindale et al., 2001, Gibson et al., 2002), we do not expect sexually antagonistic fitness variation in our pre-adult viability assays.

**Table A5.** Egg-to-pupa and pupa-to-adult viability

| **Offspring** | **No. of eggs** | **Egg-to-adult viability** | |
| --- | --- | --- | --- |
|  |  | *Egg-to-pupa percentage* | *Pupa-to-adult percentage* |
| Ho^DIDI x DIDI^ | 1580 | 67.91 | 98.42 |
| Ho^DADA x DADA^ | 1600 | 63.88 | 93.84 |
| BC^DADI X DIDI^ | 1600 | 75.63 | 98.68 |
| BC^DIDI X DADI^ | 1590 | 68.11 | 98.80 |
| F_2_^DADI X DADI^ | 1600 | 81.88 | 96.49 |

The number of developed pupa divided by the total number of eggs represents the egg-to-pupa percentage. Similarly, the number of adults emerged divided by the total number of pupa produced represents the pupa-to-adult percentage.

**Table A6. Survival values (viability) at different life stages**

| **Offspring** | **Egg-to-adult viability** | | | |  | |  |
| --- | --- | --- | --- | --- | --- | --- | --- |
|  | *Egg-to-larva*  *percentage* | | *larva-to-pupa**  *percentage* | *Pupa-to-adult*  *percentage* | | *Overall viability* | |
| Ho^DIDI x DIDI^ | 89.8 | 78.11 🡪 70,14 | | 98.42 | | 66.33 | |
| Ho^DADA x DADA^ | 75.0 | 88.88 🡪 66,66 | | 93.84 | | 57.72 | |
| BC^DADI X DIDI^ | 91.1 | 84.53 🡪 77,00 | | 98.68 | | 74.30 | |
| BC^DIDI X DADI^ | 89.9 | 78.21 🡪 70,31 | | 98.80 | | 66.91 | |
| F_2_^DADI X DADI^ | 91.2 | 90.68 🡪 82,70 | | 96.49 | | 78.37 | |

*the larva-to-pupa percentage was estimated as the difference between the pupa-to-adult viability and egg-hatchability empirically measured for each group.

We set to assess whether the offspring generated by heterokaryotypic females (BC^DADI X DIDI^ and F_2_^DADI X DADI^) have lower viability when compared to the spawn yielded by homokaryotypic females (Ho^DIDI^, Ho^DADA X DADA^, and BC^DIDI X DADI^). A Tukey’s test showed significant differences in egg-to-pupa viability between the offspring yielded by homokaryotypic and heterokaryotypic females. For the offspring produced by heterokaryotypic females, the percentage of eggs that successfully developed into pupa was significantly higher than all the other groups, contrary to the alternative hypothesis expectation of high recombination rates leading to increased embryonic and pre-adult mortality (Fig A3B). Further, a chi-square test revealed significant differences among the recombinant groups BC^DADI X DIDI^ and F_2_^DADI X DADI^ (X^2^ = 18.67; *d.f.*: 1; p < 0.001).

An analysis of covariance (ANCOVA) unveiled significant main effects of both female karyotype (F _(1,793)_ = 8.18; p = 0.004) and average genotype of the offspring (F _(1,793)_ = 4.972; p = 0.026; Table A4) on egg-to-pupa viability. As for the pupa-to-adult viability, or the proportion of pupa that emerged as adults, there were no significant differences among the tested groups (Fig A3C). Nevertheless, we found a small but significant effect of the average genotype of the offspring (F _(1,793)_ = 5.612; p = 0.018) on egg-to-adult viability. Fig A3 summarizes the offspring pre-adult viability for each juvenile stages analyzed and Tukey’s test results for each female karyotype.

**Fecundity**

For the fecundity tests, we only considered females that, at some point of the 5-day interval laid fertile eggs (that successfully developed into larvae). Our analysis did not include all females who laid non-hatching eggs exclusively, either by sterility or unsuccessful mating (McMillan et al., 1970).Table A7 summarizes the daily mean fecundity for all the crossings.

The results of the egg-to-adult viability tests showed no loss of fecundity in heterokaryotypic females for the given time interval. As summarized in Fig A4, Post hoc analysis by Tukey’s test showed that fecundity for the heterokaryotype female was significantly higher than all the other tested groups. The higher fecundity observed for heterokaryotypic individuals seems to be mostly due to the female karyotype (F _(1,188)_ = 8.88; p = 0.003; Table A4), as we found no significant effect of any of the offspring traits in our fecundity measures. Therefore, one may assume that the number of adults produced, which is the upshot of all fitness components (Bokor and Pecsenye, 2000), primarily depends on the female karyotype.

**Table A7. Daily mean fecundity (adults produced per female per day)**

| **Crossings** | | **Adult counts** | | | | | |  |  |
| --- | --- | --- | --- | --- | --- | --- | --- | --- | --- |
| Female | Male | *day 1* | *day 2* | *day 3* | *day 4* | *day 5* | *Total* | **Total Females** | **Daily mean fecundity*** |
| Ho^DIDI^ | Ho^DIDI^ | 844 | 497 | 602 | 664 | 590 | 3197 | 40 | 15.99 ±4.6 |
| Ho^DADA^ | Ho^DADA^ | 311 | 342 | 370 | 331 | 365 | 1719 | 36 | 9.55 ±4.5 |
| Ho^DIDI^ | F_1_^DADI^ | 760 | 518 | 638 | 669 | 614 | 3199 | 39 | 16.41 ±5.4 |
| F_1_^DADI^ | Ho^DIDI^ | 969 | 615 | 701 | 661 | 669 | 3615 | 38 | 19.03 ±3.7 |
| F_1_^DADI^ | F_1_^DADI^ | 639 | 635 | 611 | 997 | 867 | 3749 | 39 | 19.23 ±3.3 |

*average number of adults produced per female per day

**
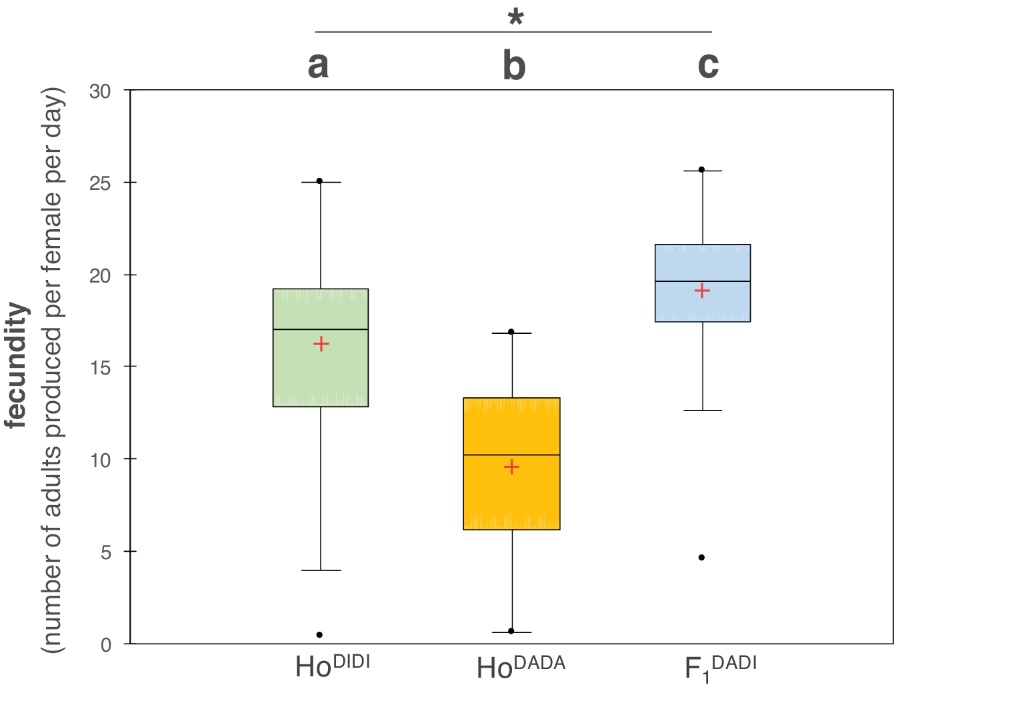
**

**Figure A4.** Fecundity for homokaryotypic (Ho ^DIDI^, Ho ^DADA^) and heterokaryotypic (F_1_^DADI^) females. The red crosses correspond to the means. Analysis of differences between karyotypes with a confidence interval of 95% (Tukey’s HSD) is displayed above each group.

The higher fecundity of heterokaryotypic females and greater viability of their offspring, might be directly related to the quality (or viability) of the eggs laid by each type. These results reject the hypothesis of high mortality of recombinant individuals in the progeny of heterokaryotypic females of *D. mediopunctata* and show that the low frequency of recombinant arrangements results from low recombination along the chromosome in inversion heterokaryotypes.

**Section 2.**

**AVERAGE GENERATION LENGTH OF *DROSOPHILA MEDIOPUNCTATA***

We obtained the temperature dataset for the period ranging from May 1999 to August 2016 from the Resende meteorologic station (Agritempo – 22º43’S, 44°45’W). To compensate for the difference in altitude between Itatiaia and the weather station (432 and 950m, respectively), we calculated the average temperature for the period considering a reduction of 6°C for each 1000m of ascent (Anslow and Shawn, 2002). Since generation length cannot be determined precisely in natural populations due to overlapping generations and reproduction at various ages (Crow and Chung, 1967), we used the period comprised between egg-to-egg stages (Djawdan et al., 1998), decomposing its two components: the egg-to-adult, and adult-to-egg time. We raised flies from the homokaryotypic strain (ITC229ET) routinely maintained in our laboratory in three different temperatures (16^o^C, 18^o^C, and 20^o^C) for at least three generations. Adult flies aged seven to ten days were crossed and transferred in sets of two couples to vials containing trimeveledon culture medium (Carvalho et al., 1989). Oviposition took place for 24 hours, and the eggs were allowed to hatch and develop at the same temperatures of their parental lines. The number of viable adults was scored every day until no more adults emerged from the vials for more than 72 hours. We computed the egg-to-adult time as the interval between egg-laying and the adult emergence peak. To determine the adult-to-egg time flies that recently emerged (within four hours or less) were transferred in sets of two couples to new vials. The pairs were then transferred to fresh vials every 24 hours. The eggs developed at the same temperatures as the parental lines. The number of adults that emerged per vial was counted. We computed the adult-to-egg time as the interval between the
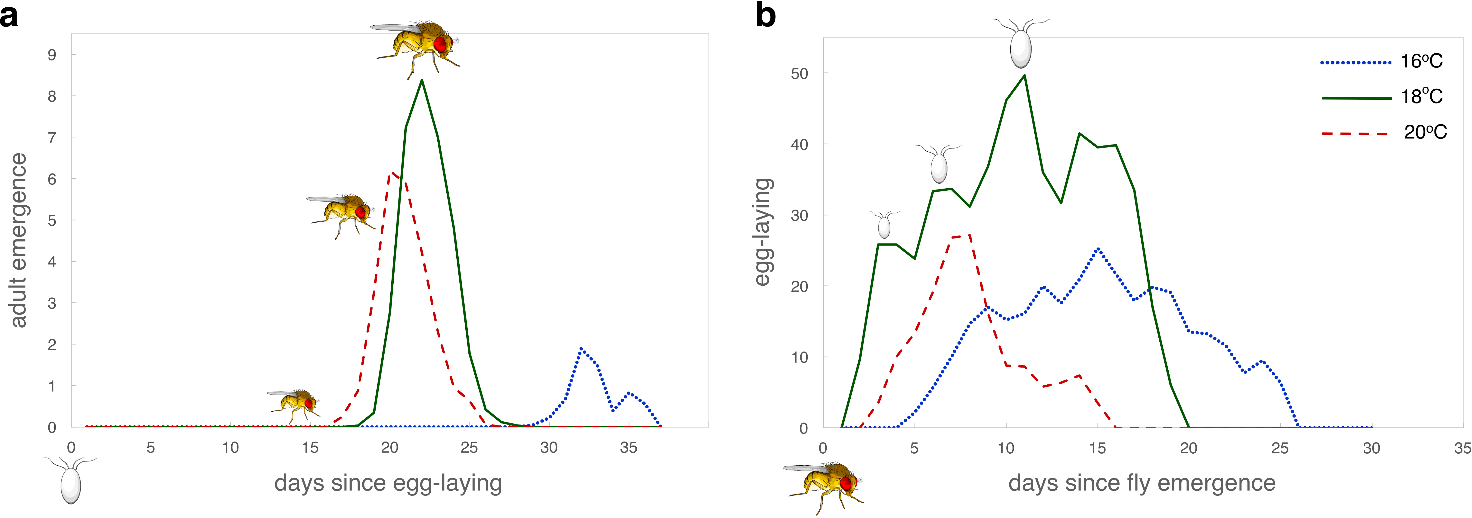
fly emergence and the peak of egg production (see Figure A5).

**Figure A5.** Average generation length of *Drosophila mediopunctata* (egg-to-egg generation time). We defined the egg-to-adult developmental time as the interval between egg-laying and the peak of fly emergence (A) and the adult-to-egg time as the period between fly emergence and the peak of egg production (B) The dotted line represents the flies reared and developed at 16^o^C, while continuous and the dashed lines denote flies reared and developed at 18^o^C and 20^o^C, respectively.

**Section 3.
SUPPLEMENTARY FIGURES**


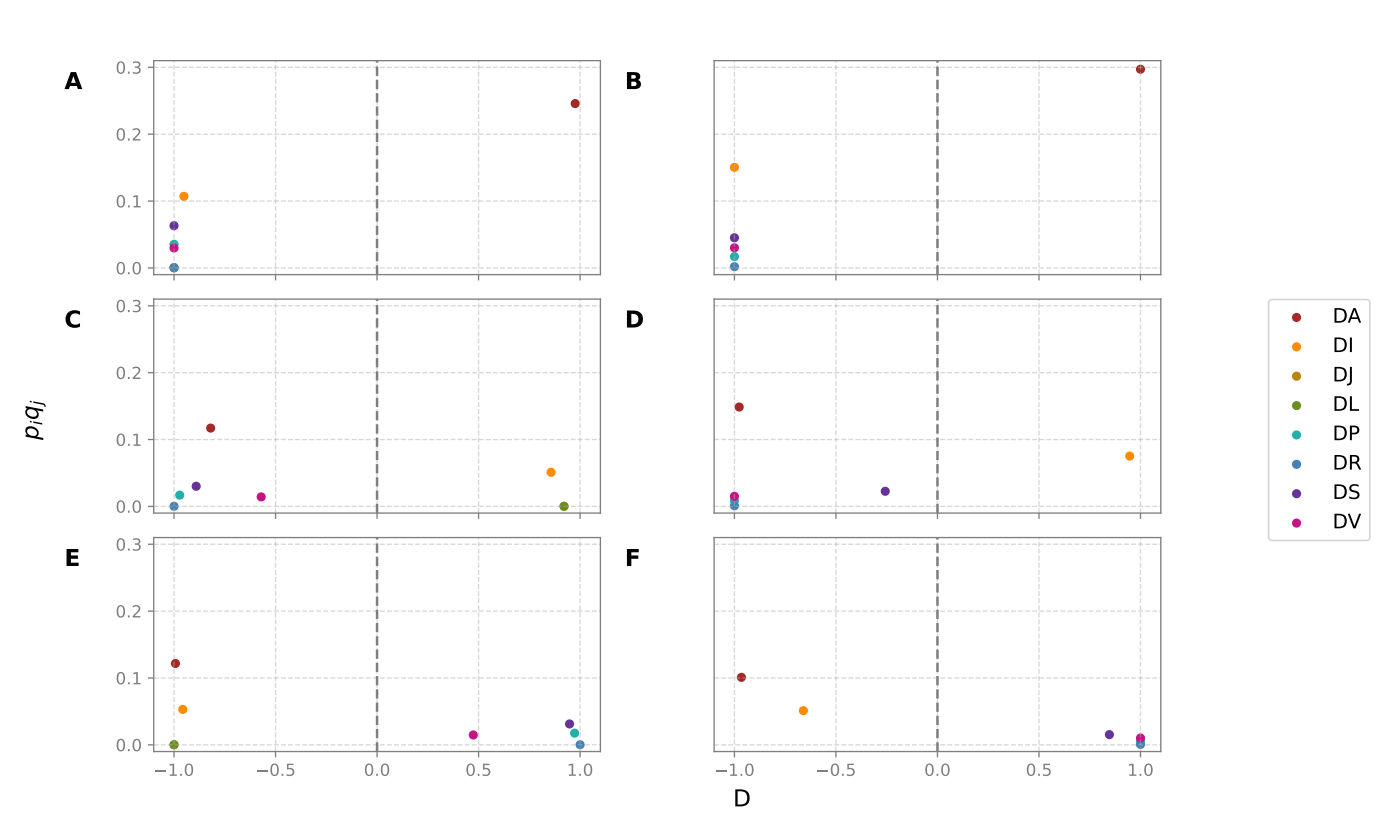


**Figure S1.** Standardized Linkage disequilibrium patterns for haplotypes associated with the proximal inversions PA0, PB0, and PC0. The left panels (A, C, E) show the patterns observed in the late 1980s (data from Peixoto and Klaczko, 1991), while the right panels (B, D, F) display the patterns observed in 2015-2016. The first row (A, B) corresponds to haplotypes associated with the PA0 inversion, the second row (C, D) with the PB0 inversion, and the third row (E, F) with the PC0 inversion.


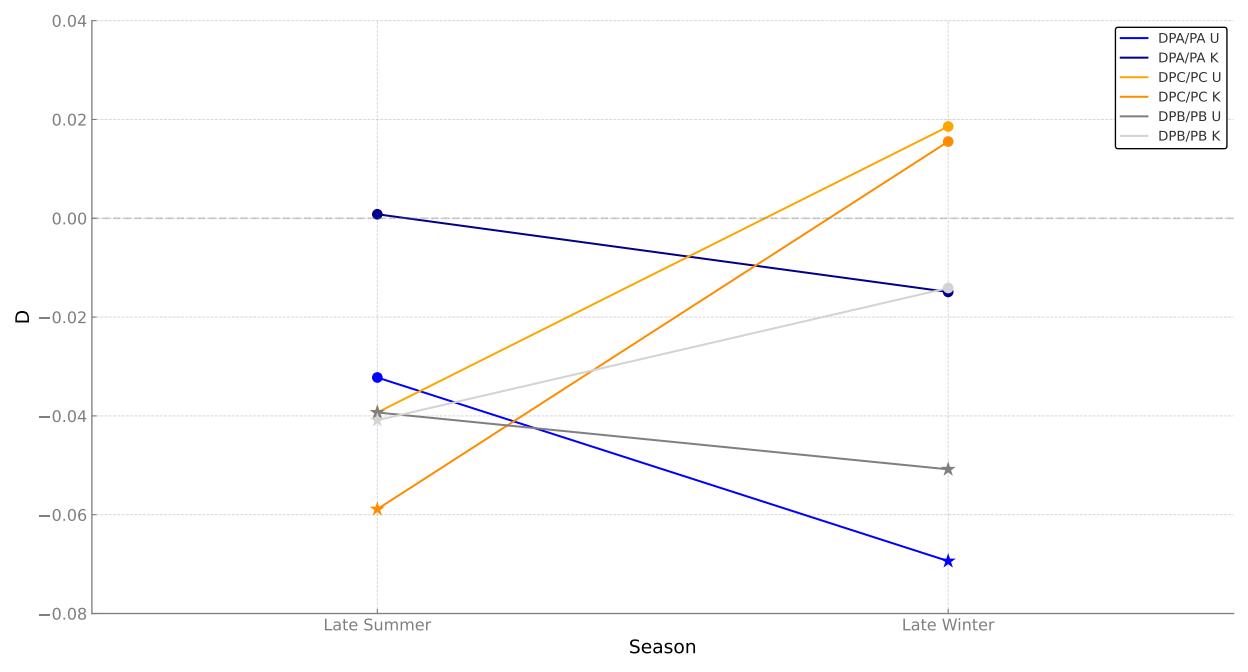


**Figure S2.** Homokaryotype frequency deviation from Hardy-Weinberg equilibrium expectation as measured by *D* (disequilibrium coefficient) for heterokaryotypes PA/PA, PC/PC and PB/PB for datasets from Klaczko et al. (1990) [K] and from collections carried out ~30 years later [U], separated by season. Stars indicate *D* values significantly different from zero.

**REFERENCES**

Anslow, F.S. and Shawn, M.J., 2002. An investigation of local alpine terrestrial lapse rates in the Canadian Rockies. *Proceedings 32nd Annual Arctic Workshop, INSTAAR*, University of Colorado, Boulder, p.1.

Bokor, K. and Pecsenye, K., 2000. Differences in the effect of ethanol on fertility and viability components among laboratory strains of *Drosophila melanogaster*. *Hereditas*, 132(3), pp.215–227.

Carvalho, A.B., Peixoto, A.A. and Klaczko, L.B., 1989. Sex-ratio in *Drosophila mediopunctata*. *Heredity (Edinb)*, 62(3), pp.425–428.

Cavasini, R., Carvalho, K.A. and Klaczko, L.B., 2010. Absence of recombination in males of *Drosophila mediopunctata*. *Drosophila Information Service*, 93, pp.122–124.

Chippindale, A.K., Gibson, J.R. and Rice, W.R., 2001. Negative genetic correlation for adult fitness between sexes reveals ontogenetic conflict in *Drosophila*. *Proceedings of the National Academy of Sciences*, 98(4), pp.1671-1675.

Coyne, J.A., Aulard, S. and Berry, A., 1991. Lack of underdominance in a naturally occurring pericentric inversion in *Drosophila melanogaster* and its implications for chromosome evolution. *Genetics*, 129(3), pp.791–802.

Crow, J.F. and Chung, Y.J., 1967. Measurement of effective generation length in *Drosophila* population cages. *Genetics*, 57, pp.951–955.

Djawdan, M., Chippindale, A.K., Rose, M.R. and Bradley, T.J., 1998. Metabolic reserves and evolved stress resistance in *Drosophila melanogaster*. *Physiological Zoology*, 71, pp.584–594.

Gibson, J.R., Chippindale, A.K. and Rice, W.R., 2002. The X chromosome is a hot spot for sexually antagonistic fitness variation. *Proceedings of the Royal Society of London. Series B: Biological Sciences*, 269(1490), pp.499-505.

Iriarte, P.F. and Hasson, E., 2000. The role of the use of different host plants in the maintenance of the inversion polymorphism in the cactophilic *Drosophila buzzatii*. *Evolution*, 54(4), pp.1295-1302.

Lande, R., 1984. The expected fixation rate of chromosomal inversions. *Evolution*, 38(4), pp.743–752.

McMillan, I., Fitz-Earle, M. and Robson, D.S., 1970. Quantitative genetics of fertility. I. Lifetime egg production of *Drosophila melanogaster* - theoretical. *Genetics*, 65(2), pp.349–353.

Pearl, R. and Parker, S.L., 1922. On the influence of density of population upon the rate of reproduction in *Drosophila*. *Proceedings of the National Academy of Sciences*, 8(7), pp.212–219.

Peixoto, A.A. and Klaczko, L.B., 1991. Linkage disequilibrium analysis of chromosomal inversion polymorphisms of *Drosophila*. *Genetics*, 129(3), pp.773–777.

Robertson, F.W. and Sang, J.H., 1944. The ecological determinants of population growth in a *Drosophila* culture. II. Circumstances affecting egg viability. *Proceedings of the Royal Society of London. Series B: Biological Sciences*, 132(868), pp.277–291.

White, M.J.D., 1969. Chromosomal rearrangements and speciation in animals. *Annual Review of Genetics*, 3(1), pp.75–98.
